# Multiple quality control checkpoints safeguard small nuclear RNA biogenesis and prevent assembly of aberrant spliceosomes

**DOI:** 10.1101/gad.353690.126

**Published:** 2026-11-01

**Authors:** Tiantai Ma, Claire Huntington, Zhuoyi Song, Rea M. Lardelli, Eric L. Van Nostrand, Jens Lykke-Andersen

**Affiliations:** 1 Department of Molecular Biology, School of Biological Sciences, University of California San Diego, La Jolla, California 92093, USA;; 2 Therapeutic Innovation Center and the Verna Marrs McLean Department of Biochemistry and Molecular Pharmacology, Baylor College of Medicine, Houston, Texas 77030, USA

**Keywords:** Integrator, NEXT–exosome, RNA quality control, TUT4, TUT7, neurodevelopmental disorders (NDD), pseudogenes, snRNA, spliceosome, splicing

## Abstract

Defective small nuclear (sn)RNAs are produced from hundreds of human snRNA pseudogenes and mutant snRNA genes associated with human developmental disorders. Machineries that prevent defective snRNAs from disrupting pre-mRNA splicing remain poorly defined. Here, we identify multiple checkpoints in snRNA biogenesis monitored by quality control machineries that subject defective snRNAs to degradation and prevent their assembly into spliceosomes. We show that variant U1 snRNAs produced from human pseudogenes, some at rates approaching canonical snRNAs, are impaired in 3′ cleavage and targeted for degradation by the NEXT–exosome while failures in subsequent protein assembly steps promote NEXT–exosome- or terminal uridylyl transferase 4/7-mediated degradation. These pathways also repress mutant snRNAs associated with human developmental disorders. Impeding snRNA quality control causes formation of aberrant spliceosomes and altered pre-mRNA splicing. These findings define checkpoints in snRNA biogenesis that safeguard pre-mRNA splicing and represent potential therapeutic targets for human disorders associated with snRNA mutations.

## Abstract (precis)

In this study, Ma et al. describe multiple quality control mechanisms that trigger the degradation of defective small nuclear RNAs (snRNAs), preventing them from assembling into aberrant spliceosomes and interfering with pre-mRNA splicing. These pseudogene-derived snRNAs or mutant snRNAs are targeted for degradation by the NEXT–exosome or TUT4/7 by faulty 3′ cleavage or snRNA-protein complex assembly, with both mechanisms serving as potentially targetable checkpoints in snRNA-related disorders.

The integrity of the pre-mRNA splicing machinery is essential for eukaryotic gene expression and aberrant splicing is associated with wide range of human disorders (Wang and Cooper 2007; Scotti and Swanson 2016). Mutations in small nuclear (sn)RNAs, the central components of the spliceosome responsible for pre-mRNA splicing, have been associated with human developmental disorders including neurodevelopmental diseases (Edery et al. 2011; He et al. 2011; Elsaid et al. 2017; Benoit-Pilven et al. 2020; Norppa and Frilander 2021; Chen et al. 2024; Greene et al. 2025; Nava et al. 2025). However, hundreds of snRNA pseudogenes exist in the human genome with the potential to produce snRNA variants containing nucleotide substitutions in residues critical for pre-mRNA splicing (Denison et al. 1981; Denison and Weiner 1982; Lund and Dahlberg 1984; O'Reilly et al. 2013; Mabin et al. 2021; Wong et al. 2024). The capacity of these snRNA variants to impact pre-mRNA splicing and the machineries that may exist to prevent their adverse effects remain poorly defined.

U1 snRNA variants in particular appear to be highly transcribed and capable of impacting pre-mRNA splicing (O'Reilly et al. 2013; Mabin et al. 2021; Wong et al. 2024). Previous studies presented evidence for association of RNA polymerase II (Pol II) with U1 snRNA pseudogenes at levels that rival those of canonical U1 snRNA genes (O'Reilly et al. 2013). Moreover, U1 snRNA variants have been observed to alter spliceosome 5′ splice site specificity when exogenously expressed in cells (Wong et al. 2024). However, U1 snRNA variants accumulate at levels at least three orders of magnitude below canonical snRNAs (Mabin et al. 2021) and those U1 snRNA variants whose stability have been directly tested were observed to be rapidly degraded (Lardelli and Lykke-Andersen 2020; Mabin et al. 2021). Thus, quality control machineries must exist that detect and degrade transcribed snRNA variants.

Defective snRNAs can also be produced from canonical snRNA genes through mutation. Recent studies have identified mutations in canonical U4-2, U2-2P and U5 snRNA genes as major drivers of neurodevelopmental disorders (NDD) (Chen et al. 2024; Greene et al. 2025; Nava et al. 2025). Moreover, mutations in minor class U4atac snRNA have been linked to developmental disorders (Edery et al. 2011; He et al. 2011; Benoit-Pilven et al. 2020; Khatri et al. 2023) and mutations in minor class U12 snRNA has been shown to cause early-onset cerebellar ataxia and CDAGS syndrome (Norppa and Frilander 2021; Xing et al. 2021). Additionally, U1 and U2 snRNAs have been reported to be mutated in multiple cancers (Shuai et al. 2019; Bousquets-Muñoz et al. 2022). Whether these disease-associated mutant snRNAs are targeted by quality control pathways remains unknown.

The biogenesis of Pol II-transcribed snRNAs, which include all spliceosomal snRNAs except U6 and U6atac, is a multistep process that involves both nuclear and cytoplasmic events (Matera and Wang 2014). Nascently transcribed snRNAs undergo cotranscriptional 7-methyl guanosine (m7G) capping followed by 3′ end cleavage by the Integrator complex (Salditt-Georgieff et al. 1980a; Baillat et al. 2005), which leaves a short snRNA 3′ end tail. The newly transcribed snRNAs are exported to the cytoplasm by export factor PHAX via interaction with the nuclear Cap-Binding Complex (CBC) bound to the m7G cap (Ohno et al. 2000). After entering the cytoplasm, snRNAs assemble with the Sm complex and are then subjected to further cap methylation to form the Tri-Methyl Guanosine (TMG) cap (Mattaj 1986; Fischer et al. 1997; Liu et al. 1997; Pellizzoni et al. 2002; Mouaikel et al. 2003). Sm complex-assembled, TMG capped snRNAs are subsequently re-imported into the nucleus (Palacios 1997; Huber et al. 1998) and subjected to nucleotide modifications by sno/scaRNAs (Bohnsack and Sloan 2018). During these nuclear and cytoplasmic biogenesis steps, snRNAs also assemble with snRNA-specific proteins, which in the case of U1 snRNA includes U1A, U1C, and U1-70K (So et al. 2016; Bohnsack and Sloan 2018; Ngu et al. 2025). One of the last steps in snRNA biogenesis involves trimming of the snRNA 3′ end tail by exonuclease TOE1 (Ma et al. 2024). Thus, nascent snRNAs for the majority of their biogenesis exist with 3′ tails that may expose them to 3′ end degradation machineries.

Here, we identify multiple checkpoints in snRNA biogenesis that are monitored by quality control pathways, which subject defective snRNA variants produced from pseudogenes and mutant snRNAs associated with human disorders for degradation from the 3′ end. These checkpoints monitor accurate 3′ end cleavage and snRNA protein complex assembly and subject defective snRNAs to degradation by the NEXT–exosome complex or terminal uridylyl transferases (TUTs) 4/7. Inactivation of the NEXT–exosome complex causes stabilization of U1 snRNA variants, assembly of U1 snRNA variants with spliceosomes, and alterations in pre-mRNA splicing. Thus, multiple snRNA biogenesis checkpoints ensure proper spliceosome assembly by preventing the accumulation of defective snRNAs and may contribute to developmental disorders by the degradation of disease-associated mutant snRNAs.

## Results

### Highly transcribed human snRNA variants are degraded during early biogenesis

The human genome harbors snRNA pseudogenes across all chromosomes (Fig. 1A). To facilitate genome-wide analyses of the fates of snRNA variants produced from pseudogenes, we created a high-throughput sequencing alignment pipeline designed to minimize faulty mapping of highly abundant reads arising from canonical snRNA genes to snRNA pseudogenes (Fig. 1B). Leveraging this pipeline, we first asked which snRNA pseudogenes are actively transcribed. Analysis of a Pol II ChIP-seq data set from human Hela cells (Beckedorff et al. 2020) showed evidence—consistent with previous studies (O'Reilly et al. 2013)—of high levels of Pol II association with a subset of snRNA pseudogenes, the majority of which were U1 snRNA pseudogenes (Fig. 1C). Furthermore, analysis of ENCODE ChIP-seq data sets for components of the snRNA gene-specific SNAPc transcription complex in human HepG2 cells similarly showed high association with a subset of snRNA pseudogenes (Supplemental Fig. S1A–C; Sadowski et al. 1993; The ENCODE Project Consortium 2012). To ask whether Pol II-associated snRNA pseudogenes could be observed to be transcribed, and if the resulting snRNA variants accumulate to the steady state, we next applied our pipelines to a HeLa cell nascent RNA-seq data set (Li et al. 2017). Indeed, a large number of snRNA variants can be observed in the nascent RNA population but are seen at much reduced levels in the steady-state population (Fig. 1D; Supplemental Table S2). This suggests that a subset of snRNA variants are highly transcribed but are rapidly degraded, consistent with previous observations for individually tested variants (Lardelli and Lykke-Andersen 2020; Mabin et al. 2021).

**Figure 1.**
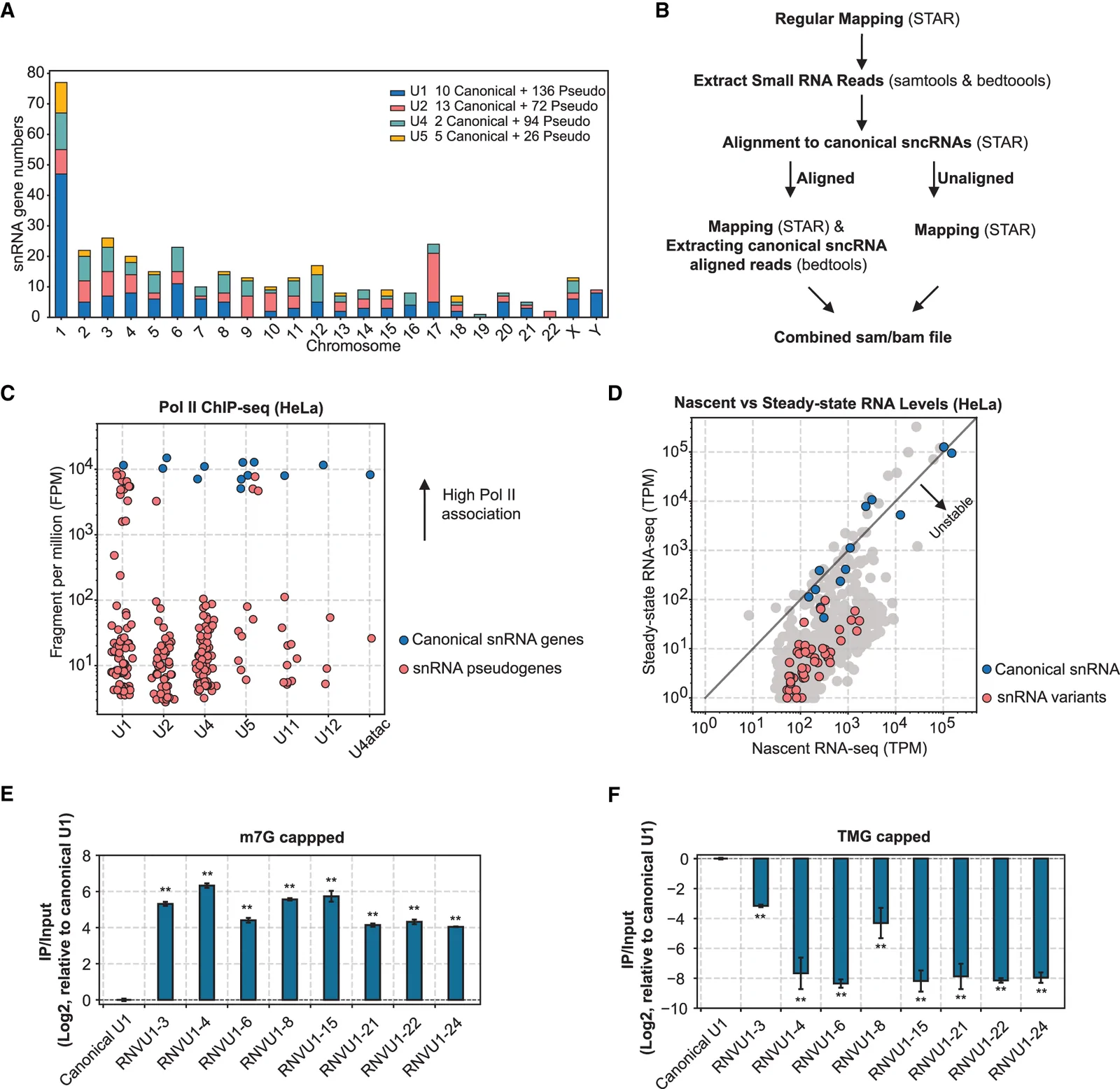
A large number of human snRNA variants are transcribed but degraded during early biogenesis. (*A*) Major class Pol II snRNA gene distribution across human chromosomes. The number of snRNA genes (GENCODE V33 annotation) is plotted for each chromosome. (*B*) Diagram of the 3-pass STAR alignment pipeline to detect snRNA variant reads from high-throughput sequencing data sets. (*C*) Scatter plot of Pol II ChIP-seq reads, represented as fragments per million (FPM), for individual Pol II snRNA canonical genes (blue) and pseudogenes (red). Data set from Beckedorff et al. (2020). See Supplemental Table S2 for canonical versus pseudogene definitions. (*D*) Scatter plot of HeLa cell nascent versus steady-state sncRNA levels by RNA seq, measured as transcripts per million reads (TPM). Canonical snRNAs are labeled in blue, snRNA variants in red, and other small noncoding RNAs in gray. Data set from Li et al. (2017). (*E*) Relative levels of m7G-capped U1 snRNA variants compared to canonical U1 snRNA, measured by m7G-cap IP followed by RT-qPCR and normalized to ratios in input RNA samples. *P*-values were calculated by Student's two-tailed *t*-test comparing variant IP/input values with those for canonical U1 snRNA (**P < 0.01). (*F*) Same as in *E* but monitoring TMG cap-IP.

To test whether snRNA variants undergo degradation during biogenesis prior to their full maturation, we took advantage of the fact that the nascent snRNA m7G cap undergoes further methylation in late biogenesis to form a TMG cap (Salditt-Georgieff et al. 1980; Mattaj 1986). Immunoprecipitation followed by RT-qPCR assays revealed that U1 snRNA variants are highly enriched for m7G-capped and de-enriched for TMG-capped species relative to canonical U1 snRNA (Fig. 1E,F). Thus, snRNA variants are degraded during biogenesis prior to maturation.

### snRNA variants are inefficiently 3′ end cleaved

An important step in early snRNA processing is cotranscriptional 3′ end cleavage by the Integrator complex, which is thought directed by a conserved 3′ box sequence occurring immediately downstream from canonical snRNA genes (Fig. 2A; Neuman de Vegvar et al. 1986; Hernandez and Lucito 1988; Uguen 2003; Baillat et al. 2005). All of the most highly Pol-II-associated U1 snRNA pseudogenes deviate from the 3′ box sequence consensus by one or more nucleotides (Fig. 2B). Canonical snRNAs that are produced with 3′ end extensions due to failure in 3′ end cleavage have been observed to undergo degradation (Hrossova et al. 2015). We therefore considered whether U1 snRNA variants are unstable due to inefficient Integrator-mediated 3′ cleavage. Indeed, metagene analyses of Pol II ChIP-seq data sets from human HeLa cells (Beckedorff et al. 2020) revealed a significant increased association of Pol II with downstream regions of U1 snRNA pseudogenes as compared to canonical U1 snRNA genes (Fig. 2C; Supplemental Fig. S2A). This pattern could be observed also for individual U1 snRNA pseudogenes (Fig. 2D). A similar analysis of a HeLa cell ChIP-seq data set for INTS11 (Beckedorff et al. 2020), the endonuclease component of the Integrator complex, shows significantly decreased association with the gene bodies of U1 snRNA pseudogenes as compared to the canonical genes (Fig. 2E,F; Supplemental Fig. S2B), suggesting impaired recruitment of INTS11 during U1 pseudogene transcription. Furthermore, analyzing an INTS11 depletion RNA-seq data set from HeLa cells (Beckedorff et al. 2020) revealed a significant increase in the accumulation of canonical snRNAs with 3′ end extensions upon INTS11 depletion, whereas snRNA variants were mostly unaffected (Fig. 2G), consistent with the idea that snRNA variants are poorly 3′ end cleaved by Integrator.

**Figure 2.**
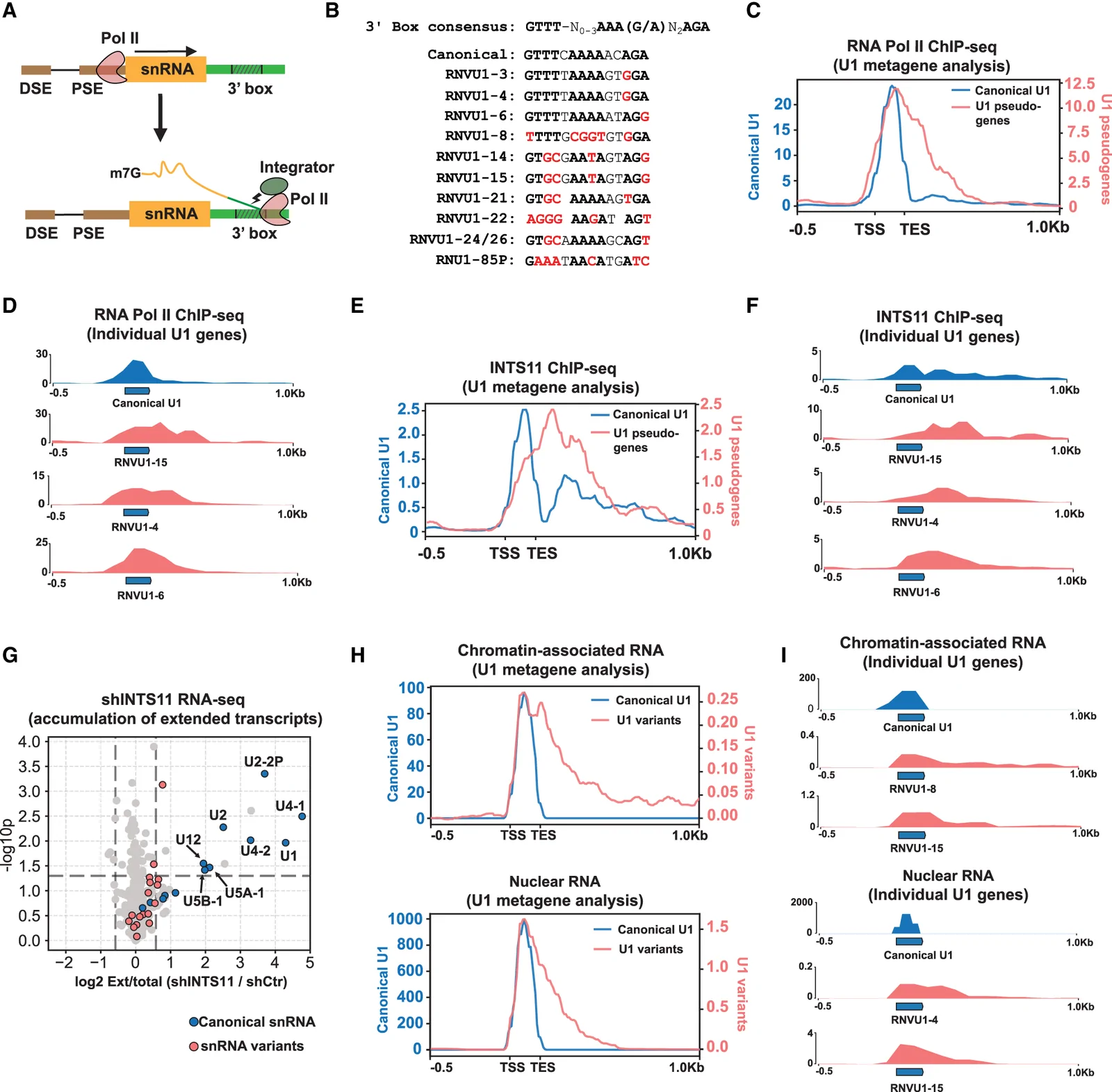
snRNA variants are inefficiently 3′ end cleaved. (*A*) Schematic of snRNA transcription by RNA Pol II. snRNA gene elements, DSE, PSE, and 3′ box, are shown at their relative snRNA gene position. Integrator cleavage is shown as a lighting symbol. (*B*) Alignment of 3′ box sequences of canonical U1 snRNA genes and U1 snRNA pseudogenes. Conserved 3′ box nucleotides are shown in bold black. Nucleotides in snRNA pseudogenes that do not match consensus are shown in bold red. (*C*) Metagene plot showing average distribution of HeLa cell Pol II ChIP-seq reads for canonical U1 genes and all transcribed U1 pseudogenes. Data set from Beckedorff et al. (2020). Values on the left represent average BPM (bins per million mapped reads) per canonical U1 gene and on the right represent average U1 pseudogene signal. Signals from 0.5 kb upstream of the transcription start site (TSS) to 1 kb downstream from the transcription end site (TES) are plotted. (*D*) Individual signal tracks for average canonical U1 genes and select U1 pseudogenes from the Pol II ChIP-seq data set in *C*. (*E*) Metagene plot as in *B* but plotting INTS11 ChIP-seq signals from Beckedorff et al. (2020). (*F*) Signal track as in *D* monitoring INTS11 ChIP-seq. (*G*) Volcano plot showing accumulation of 3′ end extended sncRNAs in INTS11 depletion (shINTS11) relative to control (shCtr) conditions, measured as the log_2_ fold ratios in shINTS11 condition over control conditions of reads 1–500 nt downstream from annotated 3′ ends over the sum of gene body and downstream TPMs. *P*-values represent three individual biological repeats calculated by Student's two-tailed *t*-test. Canonical snRNAs are labeled in blue, snRNA variants in red, and other sncRNAs in gray. (*H*) Metagene plots as in *C* for chromatin-associated (upper panel) and nuclear (lower panel) RNA read distributions. RNA-seq data sets are from Nojima et al. (2018). (*I*) Individual track plots as in *D* for chromatin-associated and nuclear RNA reads for select U1 snRNA genes.

To more directly address whether U1 snRNA variants are indeed produced with 3′ end extensions, we analyzed chromatin-associated and nuclear RNA-seq data sets from HeLa cells (Nojima et al. 2018). Indeed, a significantly larger fraction of downstream reads is observed for U1 snRNA variants in comparison to canonical U1 snRNA as observed in metagene analyses (Fig. 2H; Supplemental Fig. S2C,D) as well as in analyses of individual snRNA variants (Fig. 2I). Further verifying that transcribed snRNA variants are 3′ extended, RT-qPCR assays designed to monitor extended over total snRNA populations showed significantly higher proportions of 3′ end extended variant U1 snRNAs relative to canonical U1 snRNA (Supplemental Fig. 2E,F). Taken together, our observations show that U1 snRNA pseudogenes are defective in 3′ end cleavage and produce snRNA variants with extended 3′ ends.

### The NEXT–exosome complex degrades 3′ end extended snRNA variants

The NEXT–exosome complex is known to degrade canonical snRNAs with 3′ end extensions (Fig. 3A; Hrossova et al. 2015). To test whether snRNA variants are subjected to degradation by the NEXT–exosome, we first applied our snRNA variant analysis pipeline to an RNA-seq data set from HeLa cells in which the MTR4 RNA helicase component of the NEXT complex was depleted (Fan et al. 2017, 2018). We found that all U1 snRNA variants observed in the data set are significantly upregulated upon MTR4 depletion (Fig. 3B). Analysis of read coverage for individual transcripts demonstrated that for canonical U1 snRNAs the vast majority of reads correspond to the snRNA body, but 3′ end extended molecules can be observed to accumulate upon MTR4 depletion. In contrast, U1 snRNA variants show similar levels of upregulation of both the gene body and 3′ end extensions (Fig. 3C) consistent with poor Integrator cleavage and MTR4-dependent degradation of the 3′ end extended molecules.

**Figure 3.**
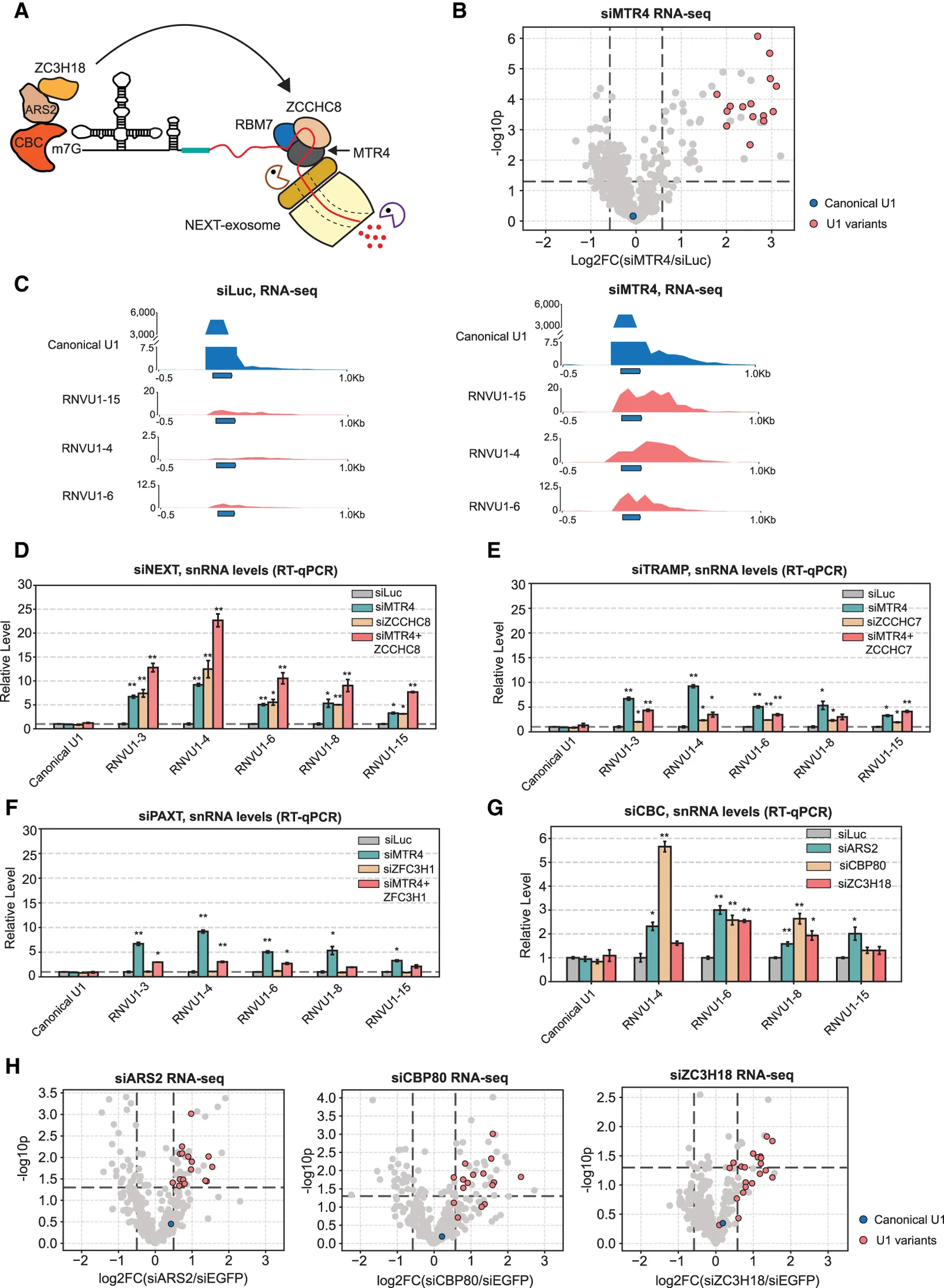
The NEXT–exosome complex degrades 3′ end extended snRNA variants. (*A*) Schematic of the NEXT–exosome complex degrading 3′ extended snRNAs stimulated by the CBC. (*B*) Volcano plot showing the log_2_ fold ratios in sncRNA levels (RNA-seq; TPM) in HeLa cells depleted for MTR4 (siMTR4) over control (siLuc). *P*-values were calculated from three individual biological repeats by Student's two-tailed *t*-test. Canonical snRNAs are labeled in blue, snRNA variants in red, and other small noncoding RNAs in gray. Data set is from Fan et al. 2017, 2018). (*C*) RNA-seq signal tracks for canonical U1 and select U1 variants in control (siLuc, *left*) and MTR4-depleted (siMTR4, *right*) conditions from the data set in *B*. Signals from 0.5 kb upstream of the transcription start site (TSS) to 1 kb downstream from the transcription end site (TES) are plotted. (*D*) Relative levels of canonical U1 and U1 variant snRNAs upon depletion of NEXT–exosome components (siNEXT represents codepletion of MTR4 and ZCCHC8) quantified by RT-qPCR and normalized to siLuc control conditions. *P*-values were calculated by two-tailed Student's *t*-tests: (*) *P* < 0.05, (**) *P* < 0.01. (*E*) Relative levels of canonical U1 and U1 variant snRNAs as in *D*, but in TRAMP-exosome depletion condition (siTRAMP represents codepletion of MTR4 and ZCCHC7). (*F*) Relative levels of canonical U1 and U1 variant snRNAs as in *D*, but in PAXT-exosome depletion condition (siPAXT represents codepletion of MTR4 and ZFC3H1). (*G*) Relative levels of canonical U1 and U1 variant snRNAs as in *D*, but in CBP80, ARS2 or ZC3H18 depletion conditions. (*H*) Volcano plots as in *B* monitoring changes in sncRNA levels upon ARS2 depletion (*left*), CBP80 depletion (*middle*), and ZC3H18 depletion (*right*) relative to control depletion conditions. Data sets are from Winczura et al. (2018).

MTR4 is the central helicase component of multiple exosome adapter complexes, including the NEXT, TRAMP and PAXT complexes (Ogami et al. 2018). To test which of these complexes is responsible for the observed U1 snRNA variant degradation we used RT-qPCR assays to monitor the impact of depletion of components specific to each of the MTR4 complexes on canonical U1 snRNA and U1 snRNA variant accumulation. We found that all tested U1 snRNA variants are significantly upregulated upon depletion of NEXT complex subunit ZCCHC8 (Fig. 3D; Supplemental Fig. S3A,B), whereas depletion of ZCCHC7 and ZFC3H1 components of TRAMP and PAXT complexes, respectively, showed only minor or no impact on accumulation of snRNA variants (Fig. 3E,F).

The NEXT–exosome complex is known to associate with the nuclear cap binding complex (CBC) via ARS2 and ZC3H18 (Fig. 3A; Andersen et al. 2013; Lubas et al. 2015; Cordiner et al. 2023; Lee et al. 2025). To address whether this association is important for snRNA variant degradation, we tested the impact of knocking down ARS2, CBP80, and ZC3H18 on U1 snRNA variant accumulation. All tested U1 snRNA variants saw increased accumulation upon depletion of one or more of these components, although some variants were more sensitive than others (Fig. 3G). Furthermore, analyses of published RNA seq data sets (Winczura et al. 2018) using our snRNA variant pipeline revealed accumulation of snRNA variants upon depletion of each of these factors (Fig. 3H), consistent with the idea that NEXT–exosome-mediated degradation of snRNA variants is functionally linked to the CBC (Fig. 3A). In contrast, depletion of the CBC-binding snRNA export factor PHAX or of the mRNA-specific CBC-binding protein NCBP3 did not significantly impact the accumulation of snRNA variants (Supplemental Fig. S3C,D). Collectively, our analyses show that snRNA variants are defective in 3′ end cleavage and targeted for degradation by the NEXT–exosome complex, stimulated by the CBC.

### Defective U1-70K binding triggers U1 snRNA degradation by the NEXT–exosome independent of a 3′ end cleavage defect

We have previously observed U1 snRNA variants with 3′ tails similar in length to Integrator-cleaved canonical snRNAs (Lardelli and Lykke-Andersen 2020; Ma et al. 2024), suggesting that a subset of snRNA variant transcription events see normal 3′ end cleavage. To monitor the fate of variant snRNAs undergoing normal 3′ end cleavage, we tested the impact of replacing the native 50 bp downstream sequence of an exogenously expressed barcoded RNVU1-15 variant (Ma et al. 2024) with the 50 bp downstream sequence of the canonical U1 snRNA *RNU1-2* gene, including the 3′ box sequence thought to direct 3′ end cleavage (Fig. 4A). Using targeted RNA sequencing, we found that this modification, as expected, resulted in significantly decreased levels of RNVU1-15 3′ extended transcripts (Fig. 4B) and significantly increased overall accumulation of RNVU1-15 RNA (Fig. 4C). However, this 3′ end-modified RNVU1-15 variant continued to accumulate at lower levels than a coexpressed canonical U1 snRNA (Fig. 4C) and remained sensitive to NEXT complex depletion (Fig. 4D), suggesting that there may be features of the RNVU1-15 variant other than 3′ end cleavage efficiency that trigger instability.

**Figure 4.**
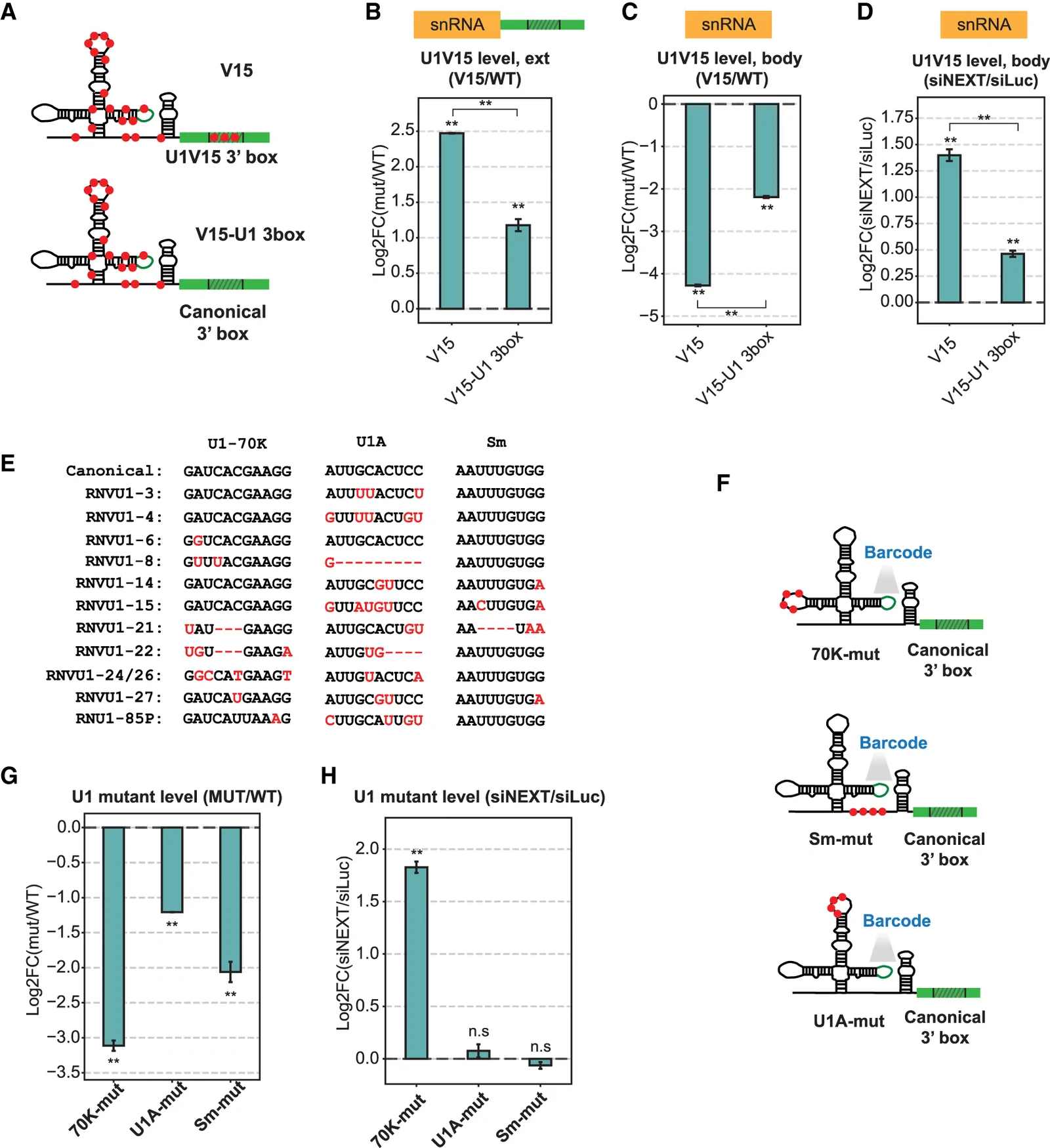
U1-70K binding site mutation triggers U1 snRNA degradation by the NEXT–exosome independent of a 3′ end cleavage defect. (*A*) Schematics of RNVU1-15 (V15; upper) and V15-U1 3box (lower) RNAs. Variant nucleotides are shown as red dots. (*B*) Log_2_ fold change in accumulation of 3′ extended V15 RNAs relative to coexpressed canonical U1 snRNA (WT), quantified by targeted RNA-seq. *P*-values comparing levels of individual extended V15 RNAs with extended WT are shown above each bar and the *P*-value for comparison of the two V15 RNAs is shown between the bars; Student's two-tailed *t*-test. (**) *P* < 0.01. (*C*) Log_2_ fold difference in total accumulation of V15 RNAs (mature plus extended) relative to coexpressed canonical U1 snRNA (WT), quantified by targeted RNA-seq. *P*-values are calculated as in *B*. (*D*) Log_2_ fold change in accumulation of V15 RNAs in NEXT depletion (siNEXT) over control (siLuc) conditions, normalized to coexpressed canonical U1 snRNA (WT), quantified by targeted RNA-seq. *P*-values are calculated as in *B*. (*E*) Sequence alignment of binding sites for U1-70K, U1A, and the Sm complex in canonical U1 and highly transcribed U1 variant snRNAs. Variant nucleotides are shown in red. (*F*) Schematics of 70K-mut, U1A-mut, and Sm-mut U1 snRNAs with mutations indicated as red dots and a barcode sequence in stem loop 3 indicated as a green line. (*G*) Log_2_ fold difference in accumulation of U1 mutant snRNAs relative to coexpressed U1 WT, quantified by targeted RNA-seq. *P*-values were calculated as in *B*. (*H*) Log_2_ fold change in U1 mutant snRNA accumulation in NEXT depletion (siNEXT) over control (siLuc) conditions quantified by targeted RNA-seq and normalized to coexpressed U1 WT. *P*-values were calculated as in *B* (n.s. *P* > 0.5).

U1 snRNA variants also harbor nucleotide variations in protein binding sites (Fig. 4E). To test whether protein assembly impacts U1 snRNA accumulation, we generated barcoded canonical U1 snRNA reporters with mutations previously established to abolish interaction with specific U1 snRNP proteins (Fig. 4F; Tsai et al. 1991; Pellizzoni et al. 2002; Hernández et al. 2009). Using targeted sequencing, we found that all tested protein assembly-deficient U1 snRNA mutants accumulate at significantly lower levels than a coexpressed unmutated U1 snRNA (Fig. 4G). This reduction in U1 snRNA accumulation is not caused by impaired 3′ end cleavage because all mutant constructs contained canonical U1 snRNA downstream sequences and produced similar levels of 3′ end extended transcripts as the coexpressed unmutated U1 snRNA (Supplemental Fig. S4). Depletion of the NEXT complex led to significant upregulation of the U1-70K binding-deficient mutant U1 snRNA, but had no effect on the other U1 snRNA mutants (Fig. 4H). Thus, the NEXT–exosome complex targets not only U1 snRNAs that fail to undergo correct 3′ cleavage but also U1 snRNA that fails to assemble with U1-70K.

### U1A and Sm complex binding site mutations trigger TUT4/7-mediated U1 snRNA degradation

Previous studies have shown that Sm-mutant U1 snRNAs are unstable (Shukla and Parker 2014; Roithová et al. 2020) and accumulate posttranscriptional oligouridine tails added by cytoplasmic terminal-uridylyl transferases (TUT) 4 and 7 (Lim et al. 2014; Roithová et al. 2020; Ma et al. 2024). We therefore next tested whether TUT4/7 depletion impacts the accumulation of U1 snRNA protein-binding mutants (Supplemental Fig. S5A,B). Indeed, mutant U1 snRNAs defective in Sm assembly and, to a less extent, U1A assembly are significantly upregulated upon TUT4/7 depletion, whereas the U1-70K assembly mutant saw no impact (Fig. 5A). Similarly, U1 snRNA engineered to contain RNVU1-15-specific variations in Sm or U1A binding sites saw increased accumulation upon TUT4/7 depletion but not upon NEXT complex depletion (Supplemental Fig. S5C–E).

**Figure 5.**
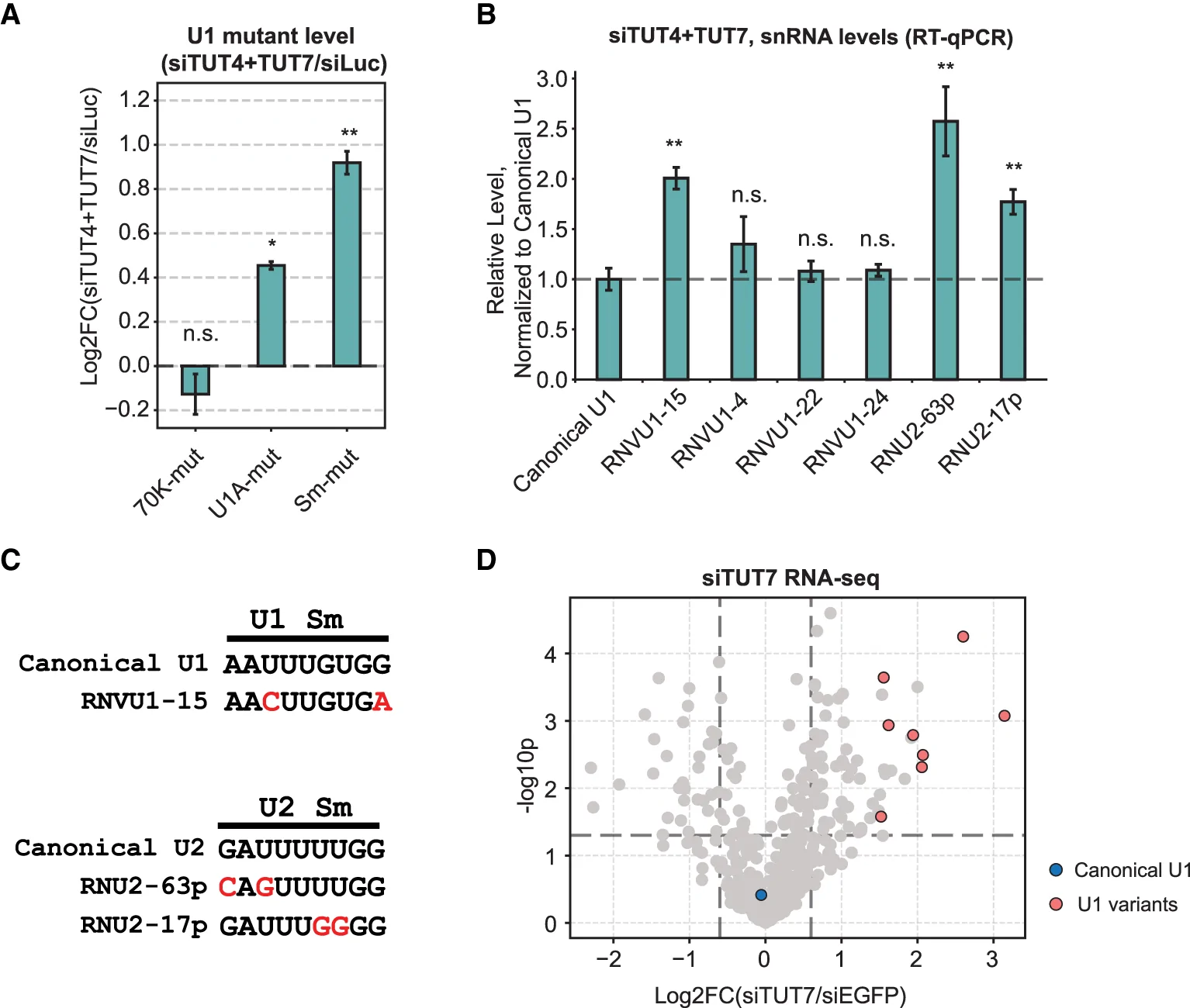
U1A and Sm complex binding site mutations trigger TUT4/7-mediated U1 snRNA degradation. (*A*) Log_2_ fold change in accumulation of U1 snRNA mutants in TUT4/7-depleted (siTUT4_7) over control (siLuc) conditions, quantified by targeted RNA-seq. *P*-values are calculated through two-tailed Student's *t*-test: (n.s.) *P* > 0.05, (*) *P* < 0.05, (**) *P* < 0.01. (*B*) Relative accumulation of canonical U1 and snRNA variants under control (siLuc) or TUT4/7-depleted (siTUT4+TUT7; using siTUT4-1 and siTUT7-1 siRNAs) conditions quantified by RT-qPCR, normalized to canonical U1. *P*-values are calculated by two-tailed Student's *t*-test. (*C*) Sequence alignment of Sm binding sites of snRNA variants upregulated under siTUT4/7 conditions. Nucleotide variations are shown in red. (*D*) Volcano plot showing the log_2_ fold change in sncRNA accumulation in TUT7-depleted (siTUT7) human PA-1 cells over control (siEGFP) conditions monitored by RNA-seq. *P*-values were calculated from three individual biological repeats by Student's two-tailed *t*-test. Canonical snRNAs are labeled in blue, snRNA variants in red, and other small noncoding RNAs in gray. Data set is from Winczura et al. (2018).

We further analyzed TUT7 and TUT4 depletion RNA-seq data sets from a human PA-1 ovarian cancer cell line (Warkocki et al. 2018). While RNVU1-15 was the only U1 snRNA variant significantly upregulated upon TUT4 depletion (Supplemental Fig. S5F), all detected U1 snRNA variants saw significant upregulation upon depletion of TUT7 (Fig. 5D). This is consistent with the fact that nearly all U1 snRNA variants harbor variations in U1A and/or Sm binding sites (Fig. 4D) and suggests that there may be some differences in which snRNA variants are targeted by TUT4 versus TUT7. RT-qPCR assays to monitor the accumulation of select endogenous snRNA variants in HeLa cells confirmed three snRNA variants, RNVU1-15, RNVU2-17P and RNVU2-63P, all of which harbor variations in the Sm binding site, as significantly upregulated following TUT4/7 depletion (Fig. 5B,C; Supplemental Fig. S5G). TUT4/7-mediated RNA uridylation is known to trigger degradation by the DCP2 decapping/XRN1 5′-exonuclease machinery (Mullen and Marzluff 2008; Rissland and Norbury 2009) as well as by 3′ exonuclease DIS3L2 (Pirouz et al. 2016; Ustianenko et al. 2016). Indeed, previous studies have implicated each of these enzymes in the degradation of mutant U1 snRNA defective in Sm complex assembly (Shukla and Parker 2014; Łabno et al. 2016; Roithová et al. 2020). However, we observed no significant increase in the accumulation of tested snRNA variants in HeLa cells upon DIS3L2, DCP2 or XRN1 depletion (Supplemental Fig. S5H–K), suggesting that they act redundantly downstream from TUT4/7 on the endogenous snRNA variants. Thus, defective U1 snRNAs are monitored by both NEXT–exosome and TUT4/7 degradation machineries, which recognize distinct snRNA features.

### snRNA variants assemble with spliceosomes upon NEXT complex depletion

Since the identified quality control checkpoints eliminate snRNA variants during snRNA biogenesis, we considered whether they are critical for preventing snRNA variants from assembling into the spliceosome. To address this question, we used enhanced (e)CLIP (Van Nostrand et al. 2016, 2020) to monitor the association of snRNA variants with spliceosome components SNRNP200 (BRR2) and PRPF8 (PRP8) in the absence or presence of NEXT complex depletion (Fig. 6A; Supplemental Fig. S6A,B). These specific spliceosome factors were selected because their antibodies have been previously verified for eCLIP (Van Nostrand et al. 2016, 2020) and, as components of the U4/U6.U5 tri-snRNP (Nguyen et al. 2015), they are known to associate with the U1 snRNP only after initiation of the pre-mRNA splicing process. Since neither PRPF8 nor SNRNP200 interact directly with U1 snRNA, we used formaldehyde- rather than UV-crosslinking to ensure capture of the entire spliceosome complex. This has been shown to be effective for enriching U1 snRNA in previous studies (Lardelli and Lykke-Andersen 2020), and we indeed observe enrichment of several snRNAs including U1, as well as of pre-mRNA introns over exons, by this method (Supplemental Fig. S6C–E).

**Figure 6.**
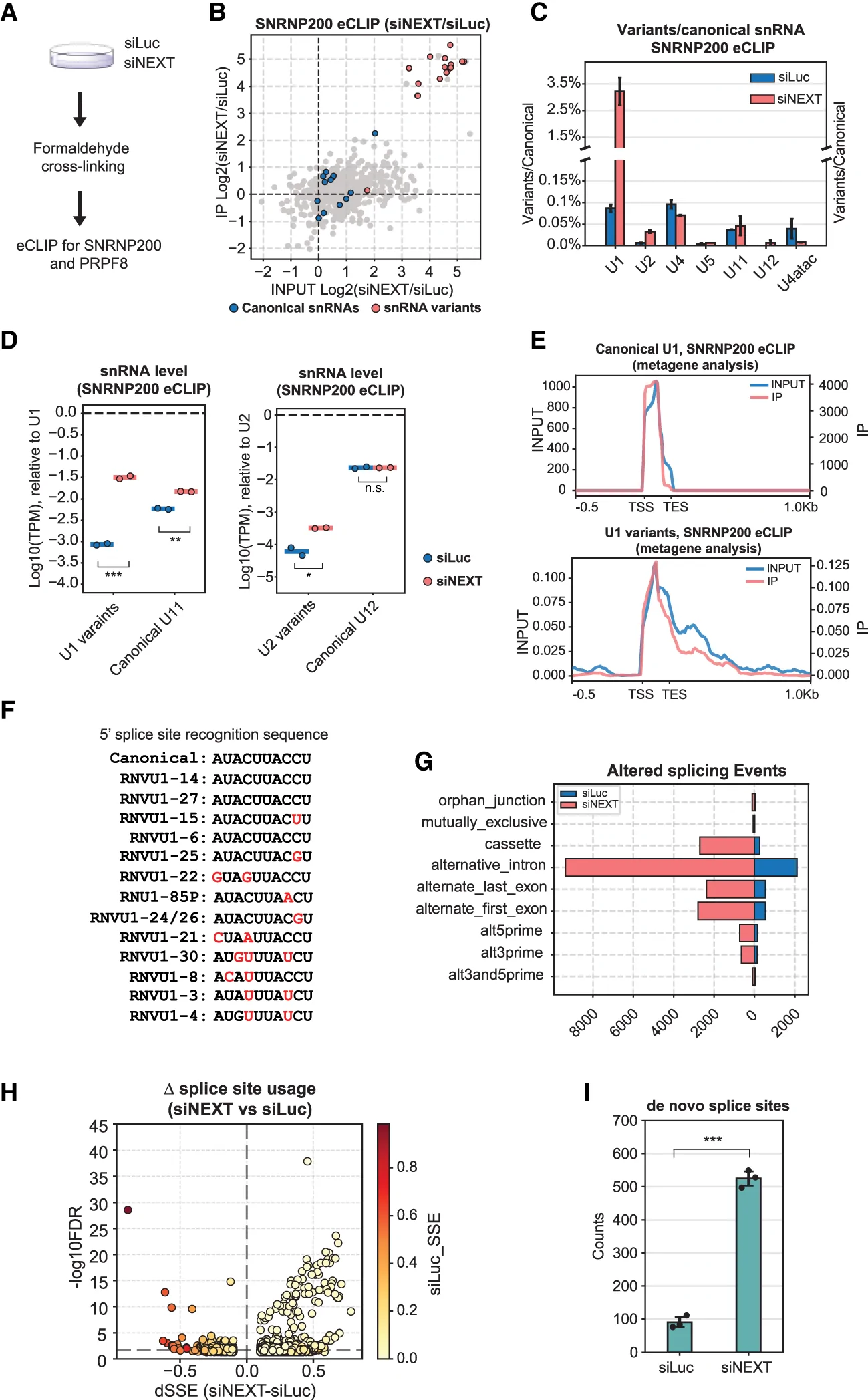
snRNA variants assemble with spliceosomes upon NEXT-complex depletion. (*A*) Schematic of eCLIP conditions. (*B*) Scatter plot of sncRNA enrichment in SNRNP200 eCLIP, showing ratios of reads (measured as TPM) in NEXT depletion (siNEXT) over control (siLuc) conditions in Input versus IP fractions. Canonical snRNAs are labeled in blue, U1 variants in red, and other sncRNAs in gray. (*C*) Bar plot showing ratios of aggregate snRNA variant levels relative to corresponding canonical snRNA levels in SNRNP200 eCLIP IP samples in siLuc (blue) and siNEXT (red) conditions. (*D*) U1 and U2 variant (each in aggregate) and canonical U11 and U12 snRNA levels relative to, respectively, canonical U1 (*left*) and U2 (*right*) snRNA levels (measured as TPM) in SNRNP200 eCLIP IP samples in siLuc (blue) and siNEXT (red) conditions. *P*-values are calculated through two-tailed Student's *t*-test: (n.s.) *P* > 0.05, (*) *P* < 0.05, (**) *P* < 0.01, (***) *P* < 0.001. (*E*) Metagene plots showing read distributions for canonical U1 snRNAs (upper panel) and U1 variants (lower panel) in SNRNP200 eCLIP input (blue) and IP (red) conditions. Signals from 0.5 kb upstream of the transcription start site (TSS) to 1 kb downstream from the transcription end site (TES) are plotted. (*F*) Sequence alignment of 5′ splice site recognition sequences in canonical U1 and U1 variant snRNAs. (*G*) Altered splicing events in eCLIP input samples, quantified by MAJIQ (Vaquero-Garcia et al. 2023), in siLuc (blue) and siNEXT (red) conditions compared to human genome annotation. (*H*) Volcano plot showing differential splice site (dSSE) usage between siNEXT and siLuc conditions in eCLIP input samples quantified by SpliSER (Dent et al. 2021). Dots represent individual splice sites colored according to fractional splice site usage in the siLuc condition (scale on the right). Only splice sites with dSSE values >0.05 or <−0.05 are shown. (*I*) Quantification of numbers of de novo splice sites observed in siLuc or siNEXT conditions in *H*. Splice sites with −log_10_(FDR) > 2 and SSE < 0.05 in opposing conditions are considered as de novo splice sites. *P*-value was calculated by two-tailed Student's *t*-test (***) *P* < 0.001.

We found that for a majority of observed U1 snRNA variants, NEXT complex depletion resulted in a 10- to 50-fold increase in their association with SNRNP200 and PRPF8 as compared with control depletion conditions (Fig. 6B; Supplemental Fig. S6F). Indeed, U1 snRNA variants were, in aggregate, observed to increase from <0.1% of the total SNRNP200- and PRPF8-associated U1 snRNA population in control conditions to 1.5%–3% (Fig. 6C; Supplemental Fig. S6G,H) upon NEXT-complex depletion, reaching levels higher than that observed for U11 snRNA of the minor spliceosome (Fig. 6D; Supplemental Fig. S6I). These observations suggest that NEXT–exosome depletion allows the assembly of spliceosomes containing variant U1 snRNAs at levels that exceed that of the minor spliceosome.

The level of U2 snRNA variants associated with SNRNP200 and PRPF8 also increased upon NEXT depletion (Fig. 6C,D; Supplemental Fig. S6H,I), mostly due to one U2 variant, RNU2-63P (Supplemental Fig. S6G). In contrast, the fraction of variants of other major and minor class snRNAs remained low and unchanged upon NEXT complex depletion, consistent with low variant expression among other snRNA classes (Fig. 1C,D). Evaluating snRNA read coverage in the eCLIP assays suggested that spliceosome assembly is selective toward variant U1 snRNAs that have undergone partial 3′ end trimming but does not require full 3′ end maturation (Fig. 6E; Supplemental Fig. S6J).

The formation of U1 snRNA variant spliceosomes upon NEXT complex depletion raised the possibility of impacts on pre-mRNA splicing. Indeed, a subset of the most abundant snRNA variants contain nucleotide variations in 5′ splice site recognition sequences (Fig. 6F), and previous studies have demonstrated that exogenous expression of U1 snRNA variants impacts 5′ splice site usage (Wong et al. 2024). Analyzing reads from our eCLIP input RNA data set, we observed a greater number of altered splicing events under NEXT depletion conditions compared to control conditions (Fig. 6G). To more specifically monitor the impact on the usage of individual splice sites, we utilized the SpliSER pipeline (Dent et al. 2021). This revealed hundreds of splice sites that saw significantly increased usage upon NEXT complex depletion, far outweighing in number the splice sites that moved in the opposite direction, and the vast majority of which were de novo sites, rarely observed in control conditions (Fig. 6H,I). Examination of representative genes seeing accumulation of mRNAs with de novo splice sites upon NEXT complex depletion confirms accumulation of alternative mRNA isoforms (Supplemental Fig. S7A). While mRNA stabilization rather than altered pre-mRNA splicing may underlie the accumulation of a subset of mRNAs with de novo splicing events, a majority were associated with genes that did not see increased transcript levels upon NEXT complex depletion (Supplemental Fig. S7B–D). Thus, consistent with previous observations of altered pre-mRNA splicing upon exogenous expression of U1 snRNA variants (Wong et al. 2024), this suggests direct alterations in splice site usage as a consequence of aberrant spliceosome assembly. Taken together, these observations suggest that the NEXT–exosome prevents the assembly of U1 snRNA variants into spliceosomes and alterations in pre-mRNA splicing.

### snRNA quality control pathways target mutant snRNAs associated with human developmental disorders

Mutations in canonical snRNA genes have been associated with developmental disorders, including neurodevelopmental disease (NDD) (Chen et al. 2024; Greene et al. 2025; Nava et al. 2025). We considered whether disease-associated mutant snRNAs, similarly to snRNA variants, may be targeted by quality control pathways monitoring snRNA biogenesis. Consistent with this idea, previous studies have observed degradation by the nuclear exosome of a mutant U12 snRNA associated with Early Onset Cerebellar Ataxia (Elsaid et al. 2017; Norppa and Frilander 2021) and reduced accumulation of a subset of mutant U4atac snRNAs associated with developmental disorders (Benoit-Pilven et al. 2020). We used exogenous expression assays to test the impact on snRNA accumulation of NDD-associated 64insU, A65G and 77insU mutations in U4-2 snRNA as well as of G48A, G51A, G55A, and U120G mutations in U4atac snRNA associated with human developmental disorders (Fig. 7A; He et al. 2011; Merico et al. 2015; Benoit-Pilven et al. 2020). When transiently expressed in HeLa cells, each of these mutant snRNAs accumulated at significantly lower levels than their coexpressed unmutated snRNA controls (Fig. 7B), although the impact of the 77insU mutation on U4-2 snRNA accumulation was minor. The LWS-associated U120G U4atac mutant, which was the most significantly downregulated snRNA mutant tested, could be observed to accumulate with an unprocessed 3′ end extension and a posttranscriptional oligo-U tail (Supplemental Fig. S8A–C).

**Figure 7.**
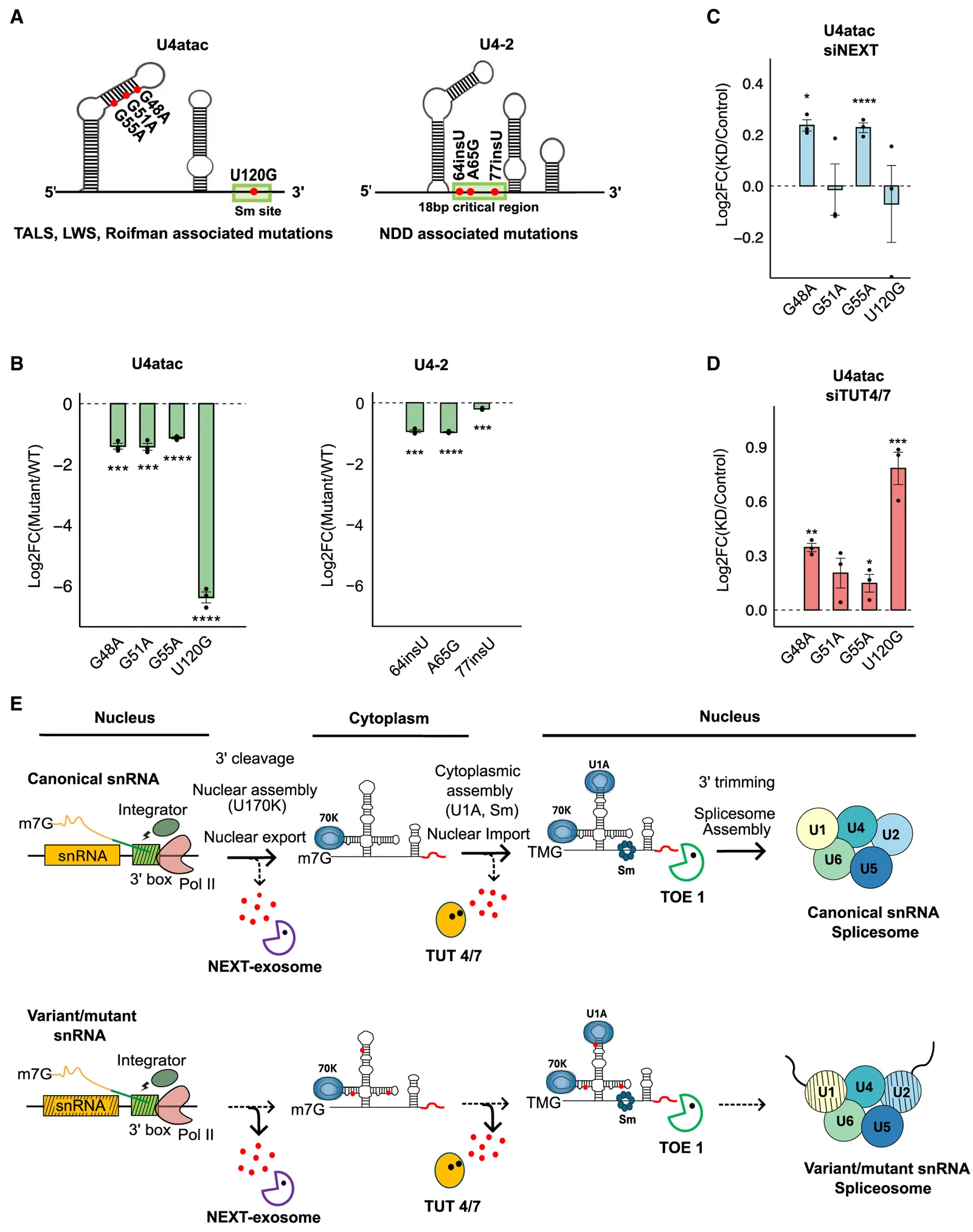
snRNA quality control pathways target mutant snRNAs associated with human developmental disorders. (*A*) Schematic of U4atac and U4-2 snRNAs highlighting tested disease-associated mutations. (*B*) Accumulation of U4atac and U4-2 mutant snRNAs relative to coexpressed WT snRNAs, quantified by targeted RNA-seq. *P*-values were calculated using two-tailed Student's *t*-test and labeled *below* the bars: (*) *P* < 0.1, (**) *P* < 0.05, (***) *P* < 0.01, (****) *P* < 0.001. (*C*) Accumulation of U4atac mutant snRNAs in siNEXT over siLuc control conditions, quantified by targeted RNA-seq and normalized to coexpressed U4atac WT snRNA. (*D*) Accumulation of U4atac mutant snRNAs in siTUT4/7 over siLuc control conditions, quantified as in *C*. (*E*) Model showing checkpoints in snRNA biogenesis monitored by quality control identified in this study and the assembly of snRNA variants/mutants into variant snRNA spliceosomes in the absence of those pathways.

To determine if the quality control pathways that target U1 snRNA variants also contribute to the observed downregulation of disease-associated mutant snRNAs, we tested the impact of depletion of the NEXT complex and TUT4/7 on the accumulation of U4atac mutant snRNAs. Depletion of the NEXT complex led to upregulation of the RFMN-associated U4atac G48A mutant and the MOPD1-associated U4atac G55A mutant, whereas the levels of U4atac G51A and U120G mutants remained unchanged (Fig. 7C). Upon depletion of TUT4/7, all tested U4atac mutants accumulated at increased levels with three of four reaching statistical significance. Collectively, these observations demonstrate the targeting of disease-associated mutant snRNAs by the same quality control pathways that target snRNA variants, with individual mutants exhibiting different sensitivities to different quality control factors.

## Discussion

The human genome harbors hundreds of snRNA pseudogenes, a subset of which are transcribed at rates approaching those of canonical snRNA genes (Fig. 1). However, the pathways that ensure that defective snRNA variants produced from pseudogenes do not interfere with normal pre-mRNA splicing and the impact of such pathways on mutant snRNAs associated with human developmental disorders have remained poorly defined. In this study, we identify multiple quality control checkpoints that monitor proper snRNA biogenesis and repress the production of defective variant snRNAs and mutant snRNAs associated with human disorders. One of these checkpoints monitors cotranscriptional 3′ end cleavage by the Integrator complex, which we demonstrate is defective for all tested U1 snRNA variants (Fig. 2), and subjects those that are aberrantly cleaved to degradation by the NEXT–exosome complex stimulated by the m7G cap-associated CBC complex (Fig. 3). Additional checkpoints monitor snRNA assembly with proteins and, in the case of U1 snRNAs, target those defective in assembly with U1-70K for degradation by the NEXT–exosome complex (Fig. 4) and those with U1A and Sm complex assembly defects for TUT4/7-mediated degradation (Fig. 5). These snRNA biogenesis checkpoints serve to prevent the formation of aberrant spliceosomes as evidenced by the assembly of U1 and U2 snRNA variants with spliceosomes and de novo splice site usage upon depletion of the NEXT–exosome (Fig. 6). The snRNA quality control checkpoints also target mutant snRNAs associated with human developmental disorders as evidenced by the negative impact of disease-associated mutations on U4-2 and U4atac snRNA accumulation and the targeting of U4atac snRNA mutants by the NEXT–exosome complex and/or TUT4/7 (Fig. 7). These findings uncover quality control checkpoints that monitor snRNA biogenesis and prevent the formation of aberrant spliceosomes containing defective snRNA variants and mutant snRNAs associated with human disorders.

Defects in U1 snRNA 3′ end cleavage and U1-70K assembly both trigger degradation by the NEXT–exosome complex, but how does the NEXT–exosome monitor each of these processes? The observations that U1 snRNA variants are stabilized upon depletion of the CBC subunit CBP80 and of factors that link the CBC to the NEXT–exosome complex demonstrate that U1 snRNA variant degradation is stimulated by the m7G cap. Previous reports have presented evidence for a kinetic competition between the snRNA nuclear export factor PHAX and the NEXT–exosome-associated protein Z3CH18 for binding to the CBC (Giacometti et al. 2017; Winczura et al. 2018; Polák et al. 2023). Moreover, the nuclear export of m7G-capped small RNAs by PHAX has been reported to be less efficient the longer the RNA (Masuyama et al. 2004). Taken together, these findings suggest that NEXT–exosome-mediated degradation of snRNA variants occurs in competition with nuclear export, such that 3′ end extended snRNA variants experience delayed nuclear export, affording the time for NEXT–exosome-mediated degradation via kinetic competition with PHAX for CBC binding (Fig. 7E). This raises the possibility that U1-70K assembly is monitored by a similar competition between nuclear export and degradation by the exosome. Indeed, U1-70K is known to promote U1 snRNA Sm complex assembly (So et al. 2016) and 3′ end trimming by TOE1 (Ma et al. 2024), consistent with the idea that U1-70K assembly occurs during early U1 snRNA biogenesis, prior to nuclear export.

Other U1 snRNA biogenesis checkpoints monitor U1A and Sm complex assembly and depend on TUT4/7-mediated degradation. The known localization of TUT4/7 (Lim et al. 2014) and of Sm complex assembly (Massenet et al. 2002) in the cytoplasm suggests that this is a cytoplasmic snRNA biogenesis checkpoint. Consistent with this idea, previous studies have implicated additional cytoplasmic decay factors, 3′ exonuclease DIS3L2 and the decapping/XRN1 5′ degradation machinery, in degrading U1 snRNAs defective in Sm binding, likely downstream from TUT4/7 (Shukla and Parker 2014; Roithová et al. 2020). The localization of U1A assembly with U1 snRNA is less well understood. Our observations suggest that U1A assembly occurs either prior to nuclear export or upon localization in the cytoplasm, either of which is compatible with a cytoplasmic quality control step. While we observed no significant upregulation upon TUT4/7 depletion of mutant U1 snRNA deficient in U1-70K binding, we have previously observed low level uridylation of this mutant snRNA (Ma et al. 2024), suggesting that a small fraction of U1-70K assembly-deficient snRNAs escape NEXT–exosome degradation and are subsequently targeted by TUT4/7, consistent with the known role of U1-70K in promoting Sm complex assembly (So et al. 2016). Sm complex assembly is known to promote TMG capping and subsequent nuclear import of snRNAs, and thus, in analogy to the competition between nuclear export and NEXT–exosome degradation, snRNA nuclear import may act in kinetic competition with TUT4/7-mediated snRNA degradation in the cytoplasm (Fig. 7E).

Our observation that U4-2 and U4atac snRNA mutations associated with developmental disorders cause snRNA downregulation raises the possibility that snRNA degradation contributes to developmental disorders associated with snRNA gene mutations. The LWS-associated U120G U4atac snRNA mutation occurs in the U4atac Sm complex binding site and, consistent with Sm binding-defective mutant U1 snRNA (Fig. 5A; Shukla and Parker 2014; Roithová et al. 2020) and U1 and U2 snRNA variants (Fig. 5B), is specifically targeted by TUT4/7 (Fig. 7D). Other tested U4atac snRNA disease mutations, which are sensitive to the NEXT complex and/or TUT4/7 (Fig. 7C,D), occur in the binding site for SNU13 (G48A) or the stem region of the 5′ stem–loop structure bound by the SNU13:PRP31 complex (G51A, G55A) (Schultz et al. 2006; Bai et al. 2024). These observations suggest that the same pathways that monitor U1 snRNA assembly also monitor distinct steps in U4atac biogenesis, including U4atac assembly with SNU13:PRP31 and the Sm complex. Similarly, mutant U12 snRNA associated with Early Onset Cerebellar Ataxia (Elsaid et al. 2017) was observed targeted by the nuclear exosome (Norppa and Frilander 2021). In addition to the U4-2 snRNA mutations tested here, mutations in U2-2P and U5 snRNAs have also been implicated in NDD (Chen et al. 2024; Greene et al. 2025; Nava et al. 2025), and evidence has been presented that U4-2 and U5 snRNA mutations lead to NDD at least in part due to specific splicing defects (Nava et al. 2025). Strikingly, recent reports have presented evidence that NDD-associated biallelic mutations in U4-2 and U2-2P snRNAs are associated with reduced snRNA accumulation, and that U4-2 mutations occur in analogous positions to U4atac mutations associated with developmental disorders (Jackson et al. 2026; Rius et al. 2026). This raises the likelihood that the snRNA quality control checkpoints identified here target a wide array of mutant snRNAs associated with human disorders. An important question for future study is to what extent downregulation via quality control checkpoints identified here contribute to the dysfunction of these disease-associated mutant snRNAs and whether interventions to promote snRNA stabilization could be a productive therapeutic avenue.

Depletion of the NEXT–exosome complex led to a dramatic increase in the association of U1 snRNA variants with spliceosomal factors SNRP200 and PRPF8 at levels above U11 snRNA of the minor spliceosome. Moreover, consistent with a subset of the most highly expressed U1 snRNA variants possessing nucleotide variation in 5′ splice site recognition sequences and with tested U1 snRNA variants having been observed to alter 5′ splice site usage when exogenously expressed (Wong et al. 2024), we observed de novo usage of hundreds of splice sites correlating with U1 snRNA variant spliceosome accumulation upon NEXT–exosome depletion. This suggests that the quality control checkpoints identified here play critical roles in safeguarding the pre-mRNA splicing process by preventing the formation of variant spliceosomes that would otherwise accumulate at levels beyond the minor spliceosome. These findings also raise the possibility that snRNA variants may become activated in certain tissues or conditions to generate specialized spliceosomes, which, in analogy with the minor spliceosome, may drive specialized splicing programs.

Our findings identify the snRNA 3′ end as a hub for quality control checkpoints that monitor snRNA biogenesis. We have previously observed that snRNAs undergo a final 3′ end trimming step by the snRNA processing enzyme TOE1 during late stages of biogenesis, after Sm complex assembly and TMG capping (Fig. 7E; Ma et al. 2024). The maintenance of an snRNA 3′ tail until the final stages of biogenesis likely allows for the multiple 3′ end-mediated quality control checkpoints identified here, highlighting the importance of competing maturation- and decay-promoting exonucleases in the quality control of small noncoding RNA biogenesis. Our findings highlight what may well be an important general principle in RNA biogenesis whereby individual processing steps are monitored by quality control checkpoints to ensure that only fully functional RNPs are allowed to accumulate in the cell, thereby preventing adverse effects of canonical RNAs that experience failures during processing and of the thousands of small noncoding RNA pseudogenes that exist in the human genome (Fig. 7E).

## Materials and methods

### Plasmids

U1 snRNA expression plasmids pU1-barcoded U1WT, pU1-70K-mut, pU1-U1A-mut, pU1-Sm-mut, pU1-v15, pU1-v15-U1A, and pU1-v15-Sm were described previously (Ma et al. 2024). Barcoded RNVU1-15 expression plasmid with a canonical U1 snRNA 3′ box (pU1-v15-U1 3 box) was generated by producing a PCR product of the barcoded RNVU1-15 sequence from pU1-v15 using V15_ligation_F and V15_overhang_R primers (Supplemental Table S1). Barcoded canonical U1 expression plasmid pU1-barcoded U1WT (Ma et al. 2024) containing canonical RNU1-2 DSE, PSE, and 3′ box sequences was linearized by polymerase chain reaction (PCR) using U1WT_bb_F and Flank_ligation_R primers (Supplemental Table S1) and ligated with the barcoded RNVU1-15 PCR product using Gibson Assembly (New England Biolabs) per manufacturer's recommendation. U4-2 and U4atac barcoded expression vectors pU4-U4-2-WT and pU4atac-U4atac-WT were assembled using HiFi DNA assembly (New England Biolabs). The expression plasmid backbone was amplified from the pU1-barcoded U1WT (Ma et al. 2024) plasmid as described above and gBlocks (IDT) containing human U4atac and U4-2 sequences with barcode mutations to allow distinction from the endogenous snRNAs (U4-2: C95G, G106C; U4atac: G94C, C102G) were assembled with the linearized plasmid; barcode mutations were designed to flip a stem–loop C-G base pair in the human snRNAs to a G-C base pair observed in other mammals. U4atac and U4-2 snRNA mutant expression plasmids (pU4atac-U4atac-G48A, pU4atac-U4atac-G51A, pU4atac-U4atac-G55A, pU4atac-U4atac-U120G, pU4-U4-2-64insU, pU4-U4-2-A65G, and pU4-U4-2-77insU) were generated in the same manner.

### Cell culture, RNA interference, and plasmid transfections

All cells were maintained in Dulbecco's modified Eagle medium (DMEM; Gibco) supplemented with 10% fetal bovine serum (FBS; Gibco) and 1% penicillin/streptomycin (Gibco) at 37°C and 5% CO_2_. RNA interference was performed in HeLa cells with 20 nM small interfering (si)RNA targeting genes of interest (Supplemental Table S1), or luciferase as a control, using siLentFect (Bio-Rad) transfection reagent according to the manufacturer's recommendations at 72 and 24 h before cell harvest. Plasmid transfections were performed using a total of 2 µg of plasmid per 3.5 cm well plates using Lipofectamine 2000 (Life Technologies) transfection reagent according to the manufacturer's recommendations at 48 h before harvest. U1 mutant plasmids were transfected in a ratio of 3:1 compared to U1WT plasmid. U4 and U4atac mutant plasmids were transfected in a ratio of 2:1 compared to U4 or U4atac WT plasmid, except for pU4atac-U4atac-U120G plasmid, which was transfected in a 4:1 ratio compared to U4atac WT plasmid.

### Targeted RNA sequencing and analysis

RNA was isolated using TRIzol (Thermo Fisher) per the manufacturer's recommendation. RNA adapters containing barcodes and 10 or 11 nt randomers (AG10/AG11) (Supplemental Table S1) were ligated to the 3′ ends of 2.5 µg of extracted RNA in a 10 µL reaction containing 1 µL of 10xT4 RNA ligase buffer (500 mM Tris-HCl pH 7.5, 100 mM MgCl_2_, 10 mM DTT), 0.2 mg/mL bovine serum albumin (BSA), 2 µM AG10 or AG11, 1 mM ATP, 10 U of T4 RNA ligase (NEB), and 40 U of RNaseOUT (Thermo Fisher) for 16 h at 16°C. Ligated RNA was subsequently treated with DNase I (1 U/µL final concentration; Zymo Research) in DNase buffer (10 mM Tris-HCl pH 7.5, 2.5 mM MgCl_2_, 0.5 mM CaCl_2_) for 15 min at 25°C, and purified by RNA isolation and concentration using an RNA Clean & Concentrator-25 kit (Zymo Research). cDNA was generated using SuperScript III (SSRT III; Thermo Fisher) in a 20 µL reaction using 0.5 µM linker-specific primer AR17 (Supplemental Table S1). Targeted RNA 3′ end sequencing libraries were generated using gene-specific forward and reverse primers (Supplemental Table S1) and sequenced on an Illumina MiSeq platform as previously described (Lardelli and Lykke-Andersen 2020; Ma et al. 2024). FASTQ files were subjected to 3′ adaptor and PCR duplicate removal using custom python scripts. The reads were mapped to a custom reference using Tailer (Nicholson-Shaw and Lykke-Andersen 2022) as previously described.

### M7G/TMG cap immunoprecipitation

Total RNA from HeLa cells was isolated using TRIzol (Thermo Fisher) per the manufacturer's recommendation. Genomic DNA was removed using a Turbo DNA-free kit (Thermo Fisher) per the manufacturer's recommendation. Twenty-five microliters of Dynabeads Protein G beads (Thermo Fisher) per sample was washed three times with NET-2 (10 mM Tris HCl pH 7.4, 150 mM NaCl, 0.1% Triton-X100), 1× protease inhibitor cocktail (Thermo Fisher), and incubated with 5 µg of anti-m7G (MBL, RN016M), anti-TMG (MBL, RN019M), or mouse IgG (Thermo Fisher 02-6300) antibody overnight at 4°C in 200 µL NET-2 with end-to-end rotation. Two micrograms of the DNA-free RNA was added to 500 µL NET-2 and 1 µL of 40 U/µL RNaseOUT (Thermo Fisher) and incubated for 5 min at 85°C and then on ice for 5 min. After heating and cooling, an additional 1 µL RNaseOUT (40 U/µL) was added to the mix. Twenty microliters of antibody-liganded Protein G beads was added to each reaction followed by incubation at 4°C with end-to-end rotation for 2 h. Beads were subsequently washed with 500 µL NET-2 eight times. After the final wash, 950 µL TRIzol was added to the beads. Immunoprecipitated RNA was subsequently isolated per the manufacturer's recommendation.

### Sequencing data analysis

FASTQ files were downloaded from NCBI Sequence Read Archive (SRA) or ENCODE Encyclopedia. Adaptors were trimmed using Cutadapt 5.1 (Martin 2011). Adaptor-trimmed reads were mapped to the human genome (version hg38) using STAR 2.7.11b (Dobin et al. 2013) in a custom three-pass alignment pipeline described in Figure 1B. Briefly, sequencing reads were first aligned to a custom FASTA database of canonical small RNA genes, each including 50 bp upstream and downstream sequences using (STAR ‐‐outFilterMultimapNmax 1000 ‐‐outFilterMultimapScoreRange 0 ‐‐outFilterMismatchNoverLmax 0.2 ‐‐outFilterMismatchNoverReadLmax 0.05 ‐‐clip5pNbases [if any] ‐‐clip3pNbases [if any] ‐‐alignIntronMin 9999999 -alignMatesGapMax 500 ‐‐alignEndsType EndToEnd ‐‐outReadsUnmapped Fastx). For ChIP-seq analysis, the first alignment step described above was skipped. Mapped (i.e., canonical sncRNA reads) and unmapped reads (i.e., reads that did not map to canonical sncRNAs) were each subsequently aligned to the full human genome using (STAR ‐‐outFilterMultimapNmax 1000 ‐‐outFilterMultimapScoreRange 0 ‐‐outFilterMismatchNoverLmax 0.025 ‐‐alignIntronMin 9999999 ‐‐alignMatesGapMax 2000 ‐‐clip5pNbases [if any] ‐‐clip3pNbases [if any] ‐‐alignEndsType EndToEnd) and, for the canonical sncRNA reads, those that did not map to canonical sncRNA genes were discarded using BEDTools (Quinlan and Hall 2010). BAM/SAM files with aligned canonical and noncanonical small RNA reads were subsequently combined for downstream analysis. Mapped reads were counted using featureCounts (Liao et al. 2014) or custom python scripts. Metagene plots were generated using deepTools (Ramírez et al. 2014). Individual gene track was generated using Integrative Genomics Viewer (IGV) 2.17.4 or using pyGenomeTracks (Lopez-Delisle et al. 2021).

### eCLIP library preparation

eCLIP was adapted from the previously described protocol (Blue et al. 2022), with modification of the crosslinking approach to stabilize indirect interactions within the spliceosome complex. Here, cells were crosslinked using 0.2% formaldehyde, followed by immunoprecipitation of spliceosomal complexes with antibodies against PRPF8 (Bethyl Laboratories A303-921A) or SNRNP200 (Bethyl Laboratories A303-453A). Two percentage of the lysate was saved as input and then denatured with 1× NuPage buffer (Life Technologies) and 500 mM DTT, separated in 4%–12% Bis-Tris SDS–polyacrylamide gels and transferred to a nitrocellulose membrane. The region corresponding to the protein size and up to the protein size plus 75 kDa was excised, and RNA was isolated following Proteinase K (NEB) treatment and column purification (Zymo). IP samples were subjected to dephosphorylation by alkaline phosphatase (FastAP, Thermo Fisher) and T4 PNK (NEB), followed by 3′ end adapter ligation (T4 RNA Ligase, NEB). After sequential low-salt and high-salt washes as in the standard eCLIP protocol, an additional wash with 2M urea buffer was performed to minimize nonspecific background. RNA was then isolated with direct Proteinase K (NEB) treatment and column purification (Zymo) (Manakov et al. 2022). Both IP and input RNA samples were then reverse transcribed and PCR-amplified according to the standard eCLIP workflow. Libraries were then sequenced on a NovaSeq X Plus (100 bp PE) platform.

### eCLIP data analysis

eCLIP data was analyzed following the previously described pipeline (Blue et al. 2022). Adapter trimmed (cutadapt v3.4) reads were mapped to the human genome using a custom STAR alignment pipeline as described above. PCR duplicates were identified and removed using UMI-tools (Smith et al. 2017) Reads were also subjected to peak calling using Clipper (Blue et al. 2022). Peaks were normalized to their paired input samples and subsequently annotated. High-confidence peaks were then identified based on filtering criteria of log_2_FC ≥ 3 and −log_10_*P* ≥ 3 and reproducibility across both biological replicates.

### qPCR assays

AR17-primed cDNA from AR17 adapter-ligated RNA described above was amplified using Fast SYBR Green master mix (Thermo Fisher) with primers for RNAs of interest (Supplemental Table S1) on a QuantStudio real-time PCR system (Thermo Fisher). Relative levels were quantified using the ΔΔ*Ct* method (Livak and Schmittgen 2001).

### Western blots

Western blots were performed by separating proteins in SDS-polyacrylamide gels followed by transfer to nitrocellulose membranes using standard procedures. Membranes were incubated overnight at 4°C with mouse polyclonal anti-ZCCHC8 (Abcam ab68739) at 1:500, rabbit polyclonal anti-MTR4 at 1:1000 (Abcam ab70551), rabbit anti-ZCCHC6 (TUT7) (Sigma HPA020615) at 1:500, rabbit anti-DCP2 (Proteintech 17744-1-AP) at 1:5000, mouse anti-H3 (Cell Signaling 14269) at 1:5000, rabbit anti-XRN1 (Lykke-Andersen and Wagner 2005) at 1:5000, and rabbit polyclonal anti-β-actin (Cell Signaling 4967S) at 1:400 each in PBS with 0.1% Tween (PBST) and 5% nonfat milk. Secondary antibodies were HPR donkey antirabbit IgG (H+L) (Thermo Fisher 31485), or HPR goat antimouse rabbit IgG (H+L) (Thermo Fisher 31430) at 1:20,000 in PBST with 5% nonfat milk. Western blots were visualized using an Odyssey Fc imaging system (LI-COR).

### Quantification and statistical analysis

Statistical tests were performed using python (v3.10.13) or R (v3.6.1). Data visualization was carried out using matplotlib (v3.8.3) or ggplot2 (v3.3.5) packages. For all experiments, appropriate statistical tests were used as described in figure legends and the Materials and Methods section.

## Supplemental Material

Supplement 1

Supplement 2

Supplement 3

Supplement 4
