# Supplementary material for: Multiple quality control checkpoints safeguard small nuclear RNA biogenesis and prevent assembly of aberrant spliceosomes
Source: Genes Dev. 2026 Nov 1;40(21-22):1837–54. doi: 10.1101/gad.353690.126 (PMC13573567; doi:10.1101/gad.353690.126)
Supplement: Supplementary file 1 [file Supplemental_Figures.pdf]

Ma_Figure S1

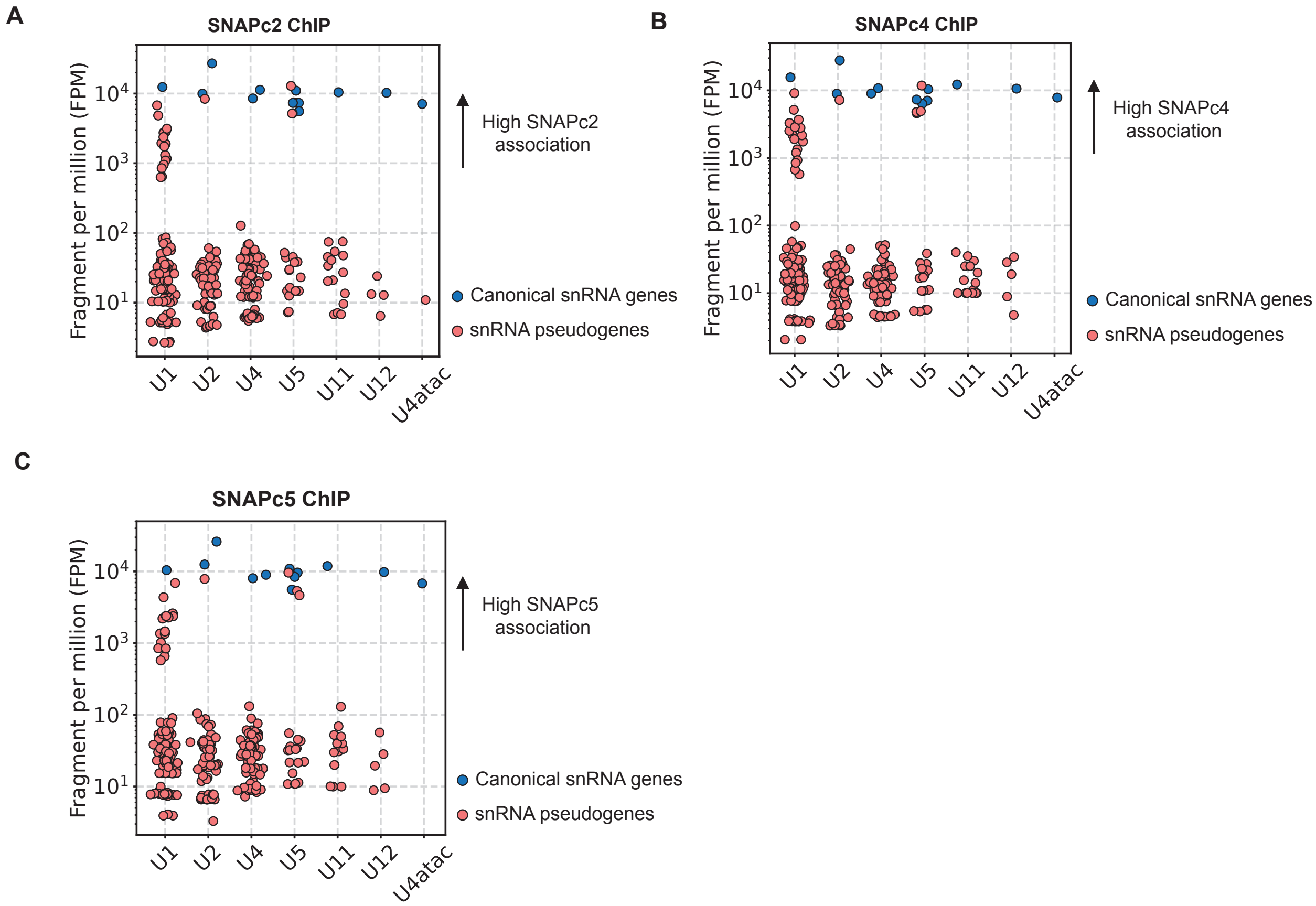

Supplementary Figure S1 related to Figure 1.

(A-C) Scatter plots of SNAPc2 (A), SNAPc4 (B), and SNAPc5 (C) ChIP seq reads, represented as fragments per million (FPM), for individual Pol II snRNA canonical genes (blue) and pseudogenes (red). Dataset from (The ENCODE Project Consortium, 2012).

Ma_Figure S2

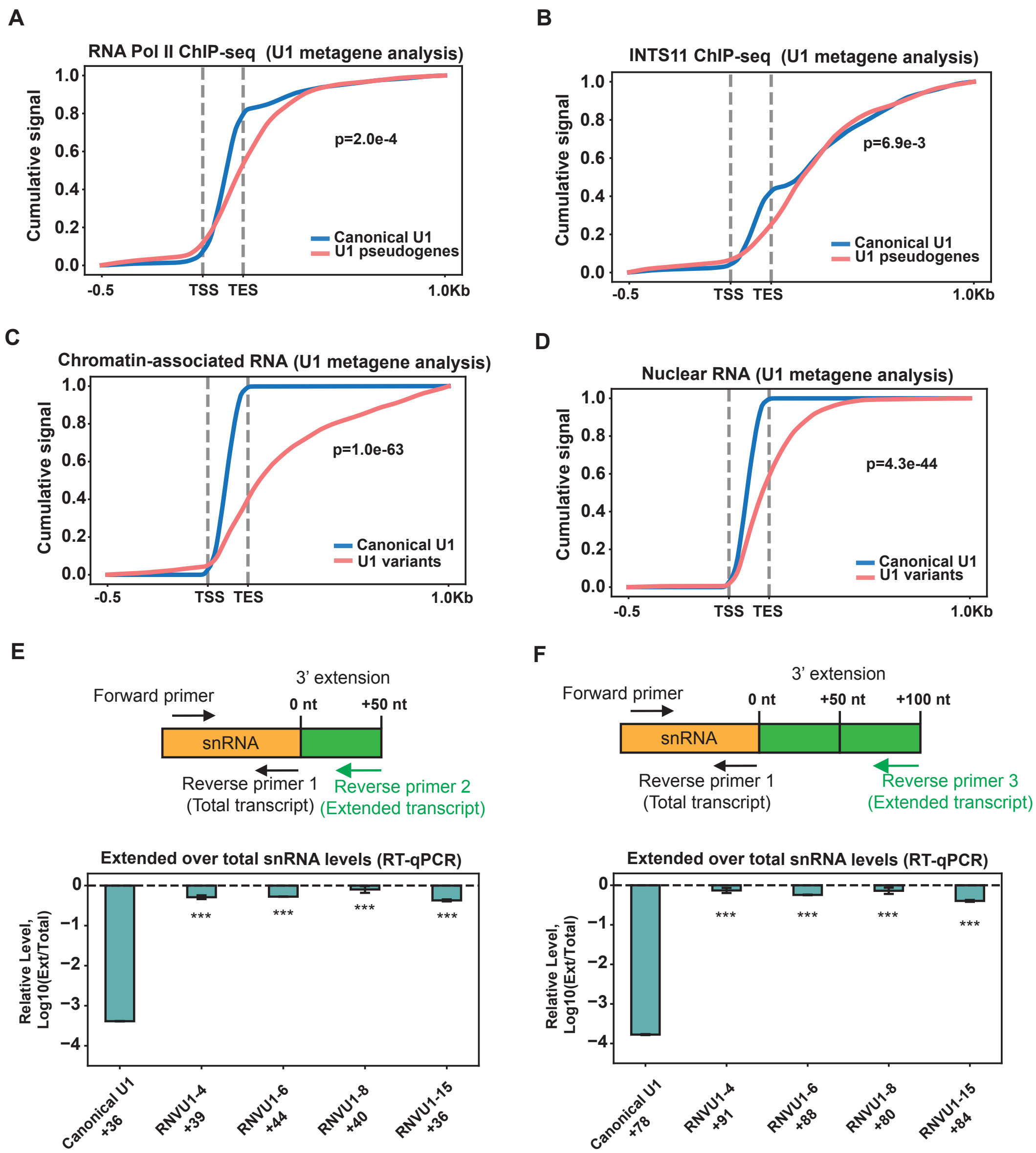
A
RNA Pol II ChIP-seq (U1 metagene analysis)
Cumulative signal
p=2.0e-4
Canonical U1
U1 pseudogenes
-0.5
TSS
TES
1.0Kb
B
INTS11 ChIP-seq (U1 metagene analysis)
Cumulative signal
p=6.9e-3
Canonical U1
U1 pseudogenes
C
Chromatin-associated RNA (U1 metagene analysis)
Cumulative signal
p=1.0e-63
Canonical U1
U1 variants
D
Nuclear RNA (U1 metagene analysis)
Cumulative signal
p=4.3e-44
Canonical U1
U1 variants
E
Forward primer
3' extension
0 nt
+50 nt
snRNA
Reverse primer 1
(Total transcript)
Reverse primer 2
(Extended transcript)
Extended over total snRNA levels (RT-qPCR)
Relative Level,
Log10(Ext/Total)
Canonical U1 +36
RNVU1-4 +39
RNVU1-6 +44
RNVU1-8 +40
RNVU1-15 +36
F
Forward primer
3' extension
0 nt
+50 nt
+100 nt
snRNA
Reverse primer 1
(Total transcript)
Reverse primer 3
(Extended transcript)
Extended over total snRNA levels (RT-qPCR)
Relative Level,
Log10(Ext/Total)
Canonical U1 +78
RNVU1-4 +91
RNVU1-6 +88
RNVU1-8 +80
RNVU1-15 +84

Supplementary Figure S2 related to Figure 2

(A) Cumulative plot showing the average cumulative signal (in BPM; Bins per million mapped reads) of HeLa cell Pol II ChIP-seq reads for Canonical U1 genes (blue) and all transcribed U1 pseudogenes (red). Dataset from (Beckedorff et al., 2020). Signals from 0.5Kb upstream of the transcription start site (TSS) to 1Kb downstream of the transcription end site (TES) are calculated and plotted. P-value is calculated using two-Sample Kolmogorov-Smirnov (KS) test.

(B) Cumulative plot as in (*A*) but plotting INTS11 ChIP-seq signal from (Beckedorff et al., 2020).

(C) Cumulative plot as in (*A*) but plotting chromatin-associated RNA signal from (Nojima et al., 2018).

(D) Cumulative plot as in (*A*) but plotting nuclear RNA signal from (Nojima et al., 2018).

(E) Log10 ratios of 3' extended snRNAs over their total snRNA populations quantified by RT-qPCR using downstream primers annealing to extended snRNA regions (Reverse Primer 2; measuring extended snRNA), starting at positions +36 to + 44 from the mature 3' end (as indicated for each variant), versus primers annealing to mature snRNA 3' end regions (Reverse Primer 1; measuring total snRNA). P-values comparing each snRNA variant to canonical U1 snRNA are calculated Student's two-tailed t-test (***: $p<0.001$)

(F) Log10 ratios of 3' extended snRNAs over their total snRNA populations as in (*E*) but using downstream primers annealing to positions between +78 and +91 as indicated.

Ma_Figure S3

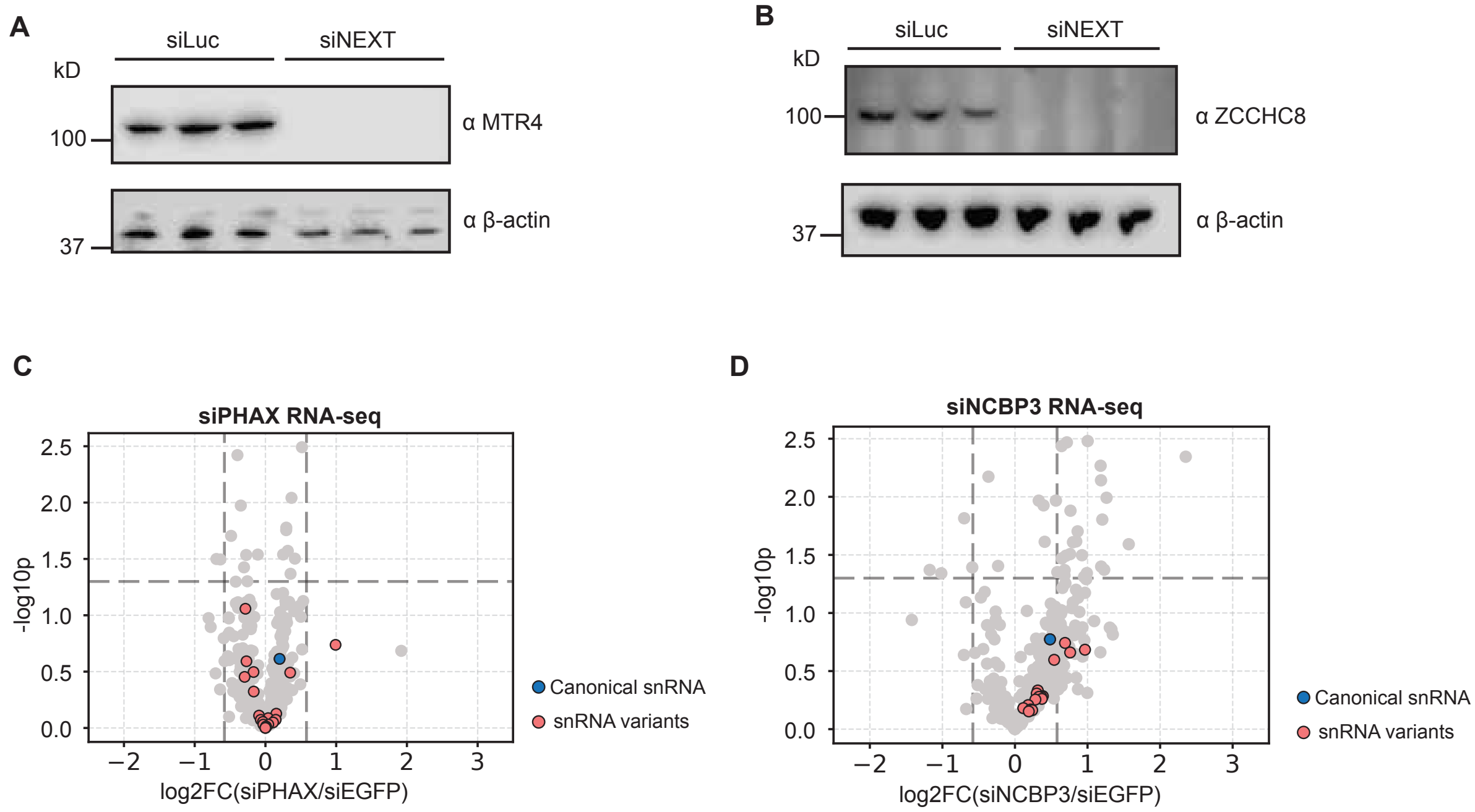

Supplementary Figure S3 related to Figure 3.
(A, B) Western blot showing depletion of MTR4 (*A*) and ZCCHC8 (*B*) in co-depletion experiments (siNEXT). β-tubulin serves as a loading control.
(C, D) Volcano plots monitoring changes in sncRNA TPMs upon PHAX depletion (*C*) and NCBP3 depletion (D) relative to control depletion conditions monitored by RNA seq. P-values were calculated from three individual biological repeats by Student's two-tailed t-test. Canonical snRNAs are labeled in blue, snRNA variants in red, and other sncRNAs in grey. Dataset is from (Winczura et al., 2018).

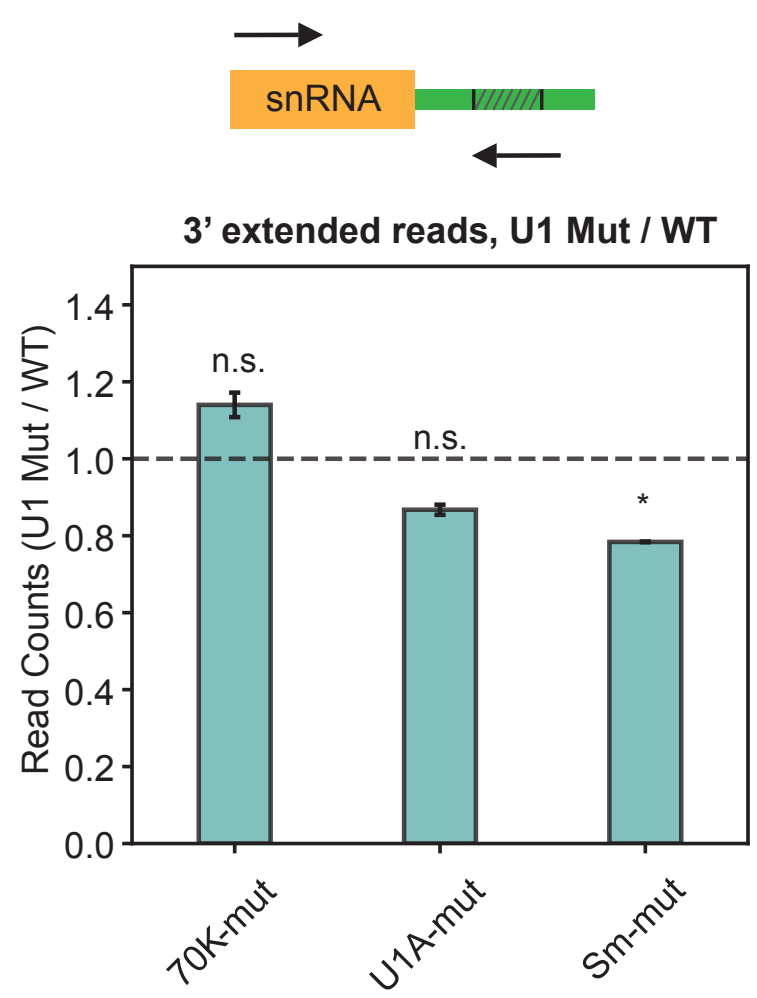

Supplementary Figure S4 related to Figure 4.
Relative levels of 3' extended 70K-mut, U1A-mut, and Sm-mut U1 snRNAs, relative to co-expressed U1 WT, quantified by targeted sequencing. P-values are calculated by two-tailed Student's t-test (n.s.: $p>0.05$, *: $p<0.05$).

Ma_Figure S5

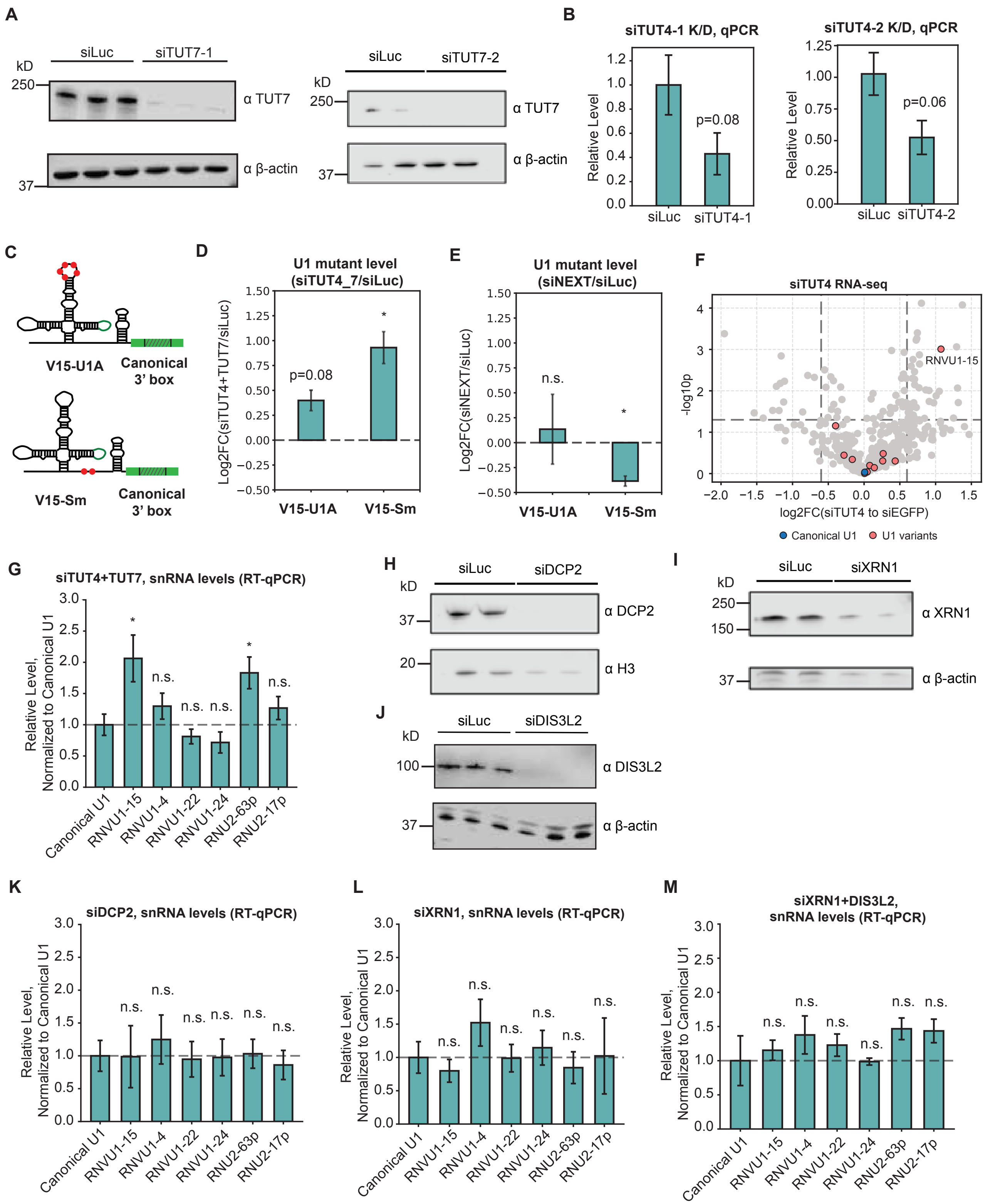
A
siLuc
siTUT7-1
kD
250
α TUT7
37
α β-actin
siLuc
siTUT7-2
kD
250
α TUT7
37
α β-actin
B
siTUT4-1 K/D, qPCR
Relative Level
p=0.08
siLuc
siTUT4-1
siTUT4-2 K/D, qPCR
Relative Level
p=0.06
siLuc
siTUT4-2
C
V15-U1A
Canonical 3' box
V15-Sm
Canonical 3' box
D
U1 mutant level (siTUT4_7/siLuc)
Log2FC(siTUT4+TUT7/siLuc)
p=0.08
*
V15-U1A
V15-Sm
E
U1 mutant level (siNEXT/siLuc)
Log2FC(siNEXT/siLuc)
n.s.
*
V15-U1A
V15-Sm
F
siTUT4 RNA-seq
-log10p
RNVU1-15
log2FC(siTUT4 to siEGFP)
Canonical U1
U1 variants
G
siTUT4+TUT7, snRNA levels (RT-qPCR)
Relative Level, Normalized to Canonical U1
*
n.s.
n.s.
n.s.
*
n.s.
Canonical U1
RNVU1-15
RNVU1-4
RNVU1-22
RNVU1-24
RNU2-63p
RNU2-17p
H
siLuc
siDCP2
kD
37
α DCP2
20
α H3
I
siLuc
siXRN1
kD
250
150
α XRN1
37
α β-actin
J
siLuc
siDIS3L2
kD
100
α DIS3L2
37
α β-actin
K
siDCP2, snRNA levels (RT-qPCR)
Relative Level, Normalized to Canonical U1
n.s.
Canonical U1
RNVU1-15
RNVU1-4
RNVU1-22
RNVU1-24
RNU2-63p
RNU2-17p
L
siXRN1, snRNA levels (RT-qPCR)
Relative Level, Normalized to Canonical U1
n.s.
Canonical U1
RNVU1-15
RNVU1-4
RNVU1-22
RNVU1-24
RNU2-63p
RNU2-17p
M
siXRN1+DIS3L2, snRNA levels (RT-qPCR)
Relative Level, Normalized to Canonical U1
n.s.
Canonical U1
RNVU1-15
RNVU1-4
RNVU1-22
RNVU1-24
RNU2-63p
RNU2-17p

Supplementary Figure S5 related to Figure 5.

(A) Western blots showing depletion of TUT7 in TUT4/TUT7 co-depletion conditions using two different sets of siRNAs. β-actin serves as a loading control.

(B) Relative levels of TUT4 mRNA in TUT4/TUT7 co-depletion conditions using two different sets of siRNAs, compared to control (siLuc) conditions, measured by RT-qPCR. Samples were normalized to the level of Mitochondrial 12S rRNA. Error bars represent SEMs from three individual experiments; p-value was calculated by Student's two-tailed t-test.

(C) Schematics of U1 snRNA mutants containing variant nucleotides from RNVU1-15 U1A (top) and Sm complex (bottom) binding sites.

(D) Accumulation of V15 mutants in siTUT4/7 (siTUT7-1 and siTUT4-1) over siLuc control conditions, quantified by targeted RNA-seq. P-values are calculated by two-tailed Student's t-test (*: $p<0.05$).

(E) Same as (*D*) but comparing siNEXT to siLuc conditions.

(F) Volcano plot showing changes in sncRNA accumulation in siTUT4 over siEGFP control conditions monitored by RNA-seq. P-values were calculated from three individual biological repeats by Student's two-tailed t-test. Canonical snRNAs are labeled in blue, variant snRNAs in red, and other sncRNAs in grey. Dataset is from (Warkocki et al., 2018).

(G) Relative accumulation of canonical U1 and snRNA variants under control (siLuc) or TUT4/7-depleted (siTUT4-2 and siTUT7-2) conditions quantified by RT-qPCR, normalized to canonical U1. P-values are calculated by two-tailed Student's t-test (*: $p<0.05$).

(H) Western blots showing depletion of DCP2. Histone H3 serves as a loading control.

(I) Western blots showing depletion of XRN1. β-actin serves as a loading control.

(J) Western blots showing depletion of DIS3L2. β-actin serves as a loading control.

(K) Relative accumulation of canonical U1 and snRNA variants as in (*G*), but in DCP2 depletion condition.

(L) Relative accumulation of canonical U1 and snRNA variants as in (*G*), but in XRN1 depletion condition.

(M) Relative accumulation of canonical U1 and snRNA variants as in (*G*), but in XRN1 and DIS3L2 co-depletion condition.

Ma_Figure S6

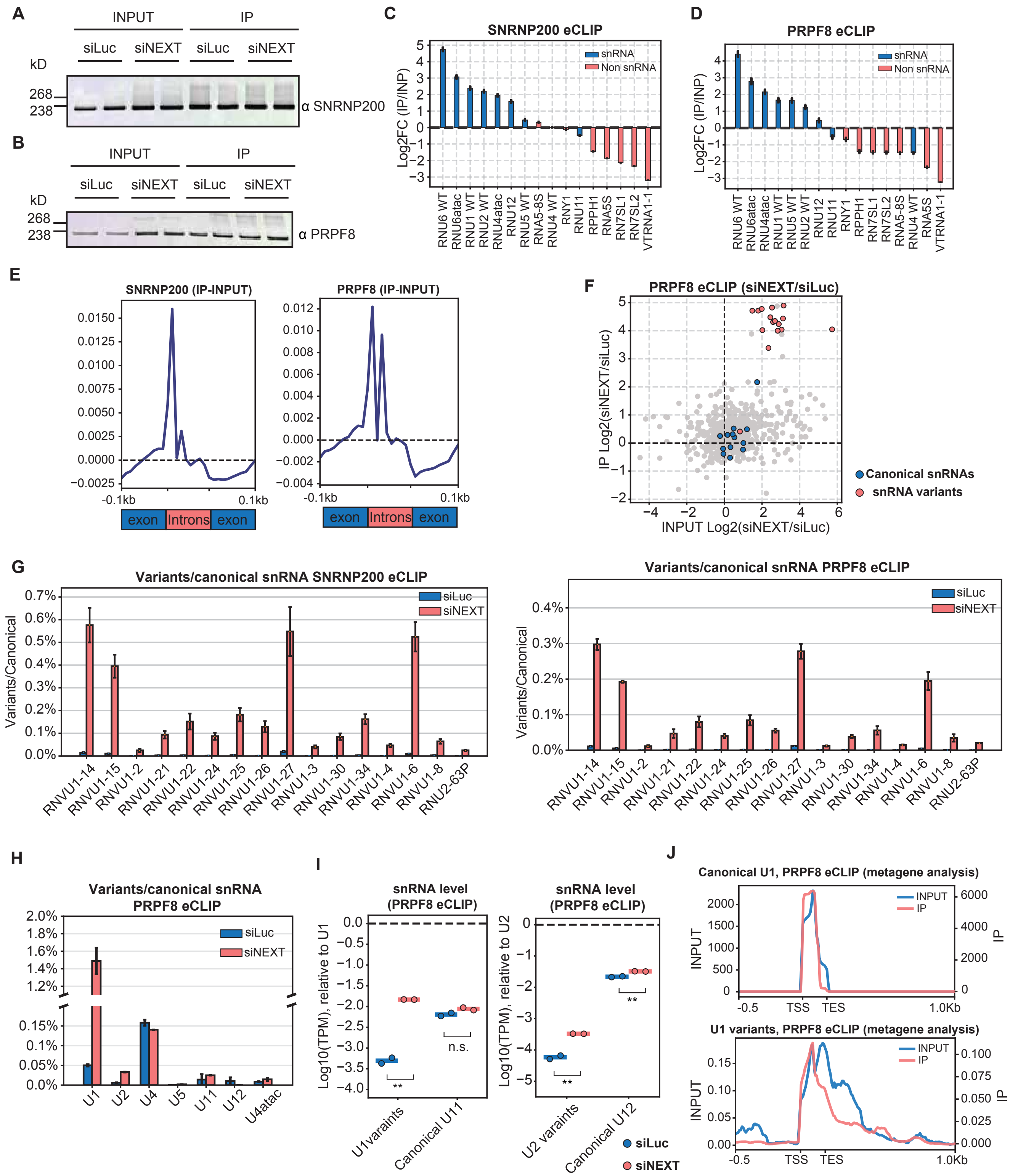

A
INPUT
IP
siLuc
siNEXT
siLuc
siNEXT
kD
268
238
α SNRNP200
B
INPUT
IP
siLuc
siNEXT
siLuc
siNEXT
kD
268
238
α PRPF8
C
SNRNP200 eCLIP
snRNA
Non snRNA
Log2FC (IP/INP)
RNU6 WT
RNU6atac
RNU1 WT
RNU2 WT
RNU4atac
RNU12
RNU5 WT
RNA5-8S
RNU4 WT
RNY1
RNU11
RPPH1
RNA5S
RN7SL1
RN7SL2
VTRNA1-1
D
PRPF8 eCLIP
snRNA
Non snRNA
Log2FC (IP/INP)
RNU6 WT
RNU6atac
RNU4atac
RNU1 WT
RNU5 WT
RNU2 WT
RNU12
RNU11
RNY1
RPPH1
RN7SL1
RN7SL2
RNA5-8S
RNU4 WT
RNA5S
VTRNA1-1
E
SNRNP200 (IP-INPUT)
PRPF8 (IP-INPUT)
-0.1kb
0.1kb
exon
Introns
exon
F
PRPF8 eCLIP (siNEXT/siLuc)
IP Log2(siNEXT/siLuc)
INPUT Log2(siNEXT/siLuc)
Canonical snRNAs
snRNA variants
G
Variants/canonical snRNA SNRNP200 eCLIP
Variants/canonical snRNA PRPF8 eCLIP
Variants/Canonical
siLuc
siNEXT
RNVU1-14
RNVU1-15
RNVU1-2
RNVU1-21
RNVU1-22
RNVU1-24
RNVU1-25
RNVU1-26
RNVU1-27
RNVU1-3
RNVU1-30
RNVU1-34
RNVU1-4
RNVU1-6
RNVU1-8
RNU2-63P
H
Variants/canonical snRNA
PRPF8 eCLIP
siLuc
siNEXT
U1
U2
U4
U5
U11
U12
U4atac
I
snRNA level
(PRPF8 eCLIP)
Log10(TPM), relative to U1
Log10(TPM), relative to U2
U1variants
Canonical U11
U2 varaints
Canonical U12
n.s.
siLuc
siNEXT
J
Canonical U1, PRPF8 eCLIP (metagene analysis)
U1 variants, PRPF8 eCLIP (metagene analysis)
INPUT
IP
-0.5
TSS
TES
1.0Kb

Supplementary Figure S6 related to Figure 6.
(A, B) Western blots for SNRNP200 (*A*) and PRPF8 (*B*) from eCLIP input and IP conditions.
(C, D) Enrichment of snRNAs (blue) and representative other sncRNAs (red) in IP relative to input conditions in SNRNP200 (*C*) and PRPF8 eCLIP (*D*).
(E) Metagene plot showing read distributions in eCLIP IP samples subtracted by read distributions in input samples over all annotated human introns and 0.1kb of flanking exons in aggregation. Introns are scaled to 0.1kb in length.
(F) Scatter plot of small non-coding RNA enrichment in PRPF8 eCLIP, showing Log2 ratios of reads (measured as TPM) in siNEXT over siLuc conditions in Input versus IP fractions. Canonical snRNAs are labeled in blue, U1 variants in red and other sncRNAs in grey.
(G) Bar plot of ratios of individual snRNA variants relative to corresponding canonical snRNAs in SNRNP200 (left) or PRPF8 (right) eCLIP IP samples in siLuc (blue) or siNEXT (red) conditions.
(H) Bar plot of the aggregate ratio of snRNA variants relative to corresponding canonical snRNAs in PRPF8 eCLIP IP samples in siLuc (blue) and siNEXT (red) conditions.
(I) U1 variant (in aggregate) and canonical U11 snRNA levels relative to canonical U1 snRNA levels (left) (measured as TPM) or aggregate U2 variants and canonical U12 snRNA relative to canonical U2 snRNA (right) in PRPF8 eCLIP IP samples in siLuc (blue) and siNEXT (red) conditions. P-values are calculated through two-tailed Student's t-test (n.s.: $p>0.05$, **: $p<0.01$).
(J) Metagene plots showing read distributions for canonical U1 snRNA (upper panel) and U1 variants (lower panel) in PRPF8 eCLIP input (blue) and IP (red) conditions. Signals from 0.5Kb upstream of the transcription start site (TSS) to 1Kb downstream of the transcription end site (TES) are plotted.

Ma_Figure S7

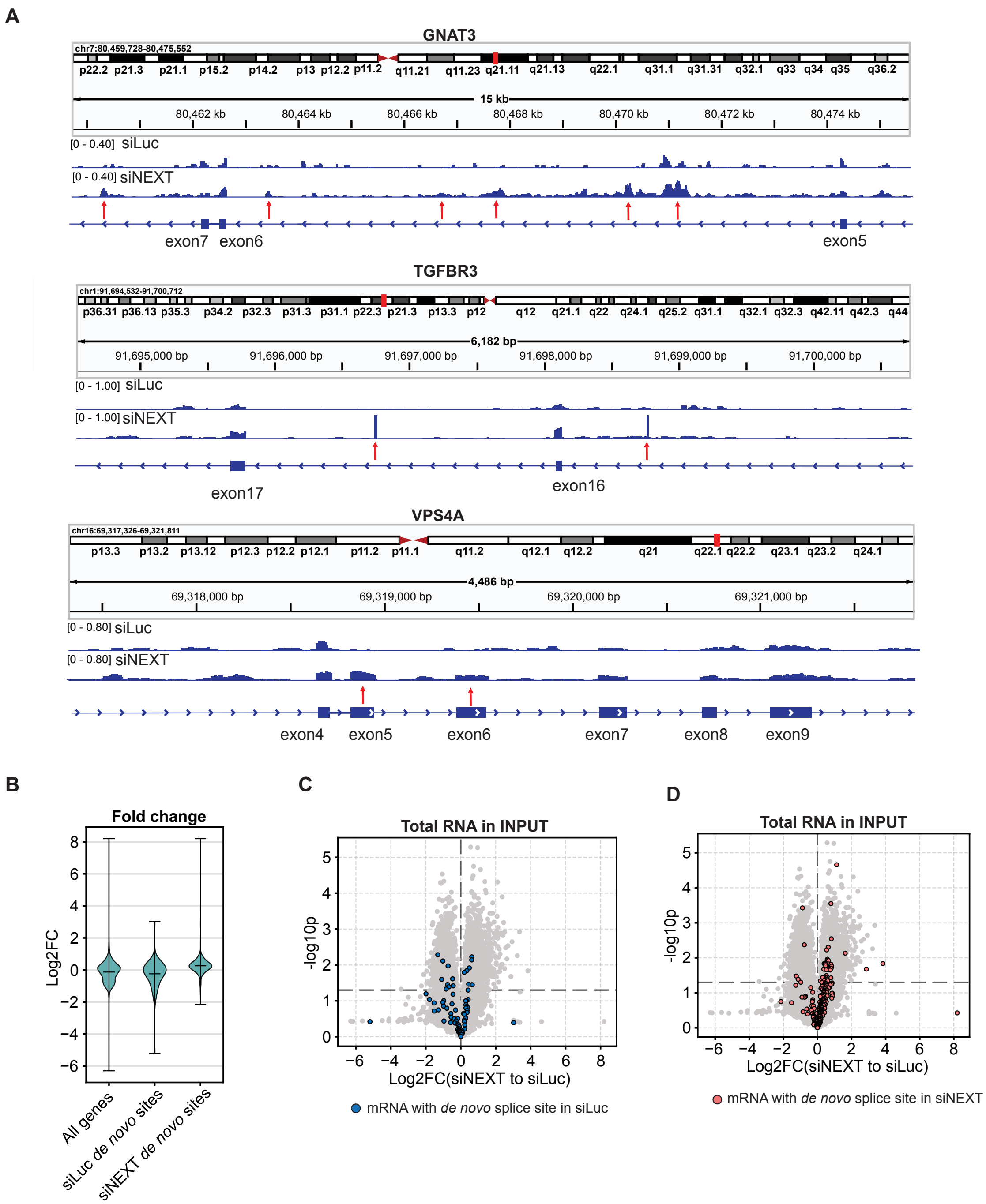

A
GNAT3
chr7:80,459,728-80,475,552
15 kb
80,462 kb
80,464 kb
80,466 kb
80,468 kb
80,470 kb
80,472 kb
80,474 kb
[0 - 0.40] siLuc
[0 - 0.40] siNEXT
exon7
exon6
exon5
TGFBR3
chr1:91,694,532-91,700,712
6,182 bp
91,695,000 bp
91,696,000 bp
91,697,000 bp
91,698,000 bp
91,699,000 bp
91,700,000 bp
[0 - 1.00] siLuc
[0 - 1.00] siNEXT
exon17
exon16
VPS4A
chr16:69,317,326-69,321,811
4,486 bp
69,318,000 bp
69,319,000 bp
69,320,000 bp
69,321,000 bp
[0 - 0.80] siLuc
[0 - 0.80] siNEXT
exon4
exon5
exon6
exon7
exon8
exon9
B
Fold change
Log2FC
All genes
siLuc de novo sites
siNEXT de novo sites
C
Total RNA in INPUT
-log10p
Log2FC(siNEXT to siLuc)
mRNA with de novo splice site in siLuc
D
Total RNA in INPUT
-log10p
Log2FC(siNEXT to siLuc)
mRNA with de novo splice site in siNEXT

Supplementary Figure S7 related to Figure 6.
(A) Individual gene track plots representing altered splice site usage in siNEXT compared to siLuc conditions. Regions showing altered splicing are labelled by red arrows.
(B) Violin plots showing relative level changes in siNEXT versus siLuc conditions of mRNA from all genes, genes with *de novo* splice sites in the siLuc condition, and genes with *de novo* splice sites in the siNEXT condition.
(C, D) Differential expression in siNEXT over siLuc conditions of mRNAs from genes with *de novo* splice sites observed in siLuc conditions (*C*, blue) or in siNEXT conditions (*D*, red) (see Figure 6I), compared to all genes (grey).

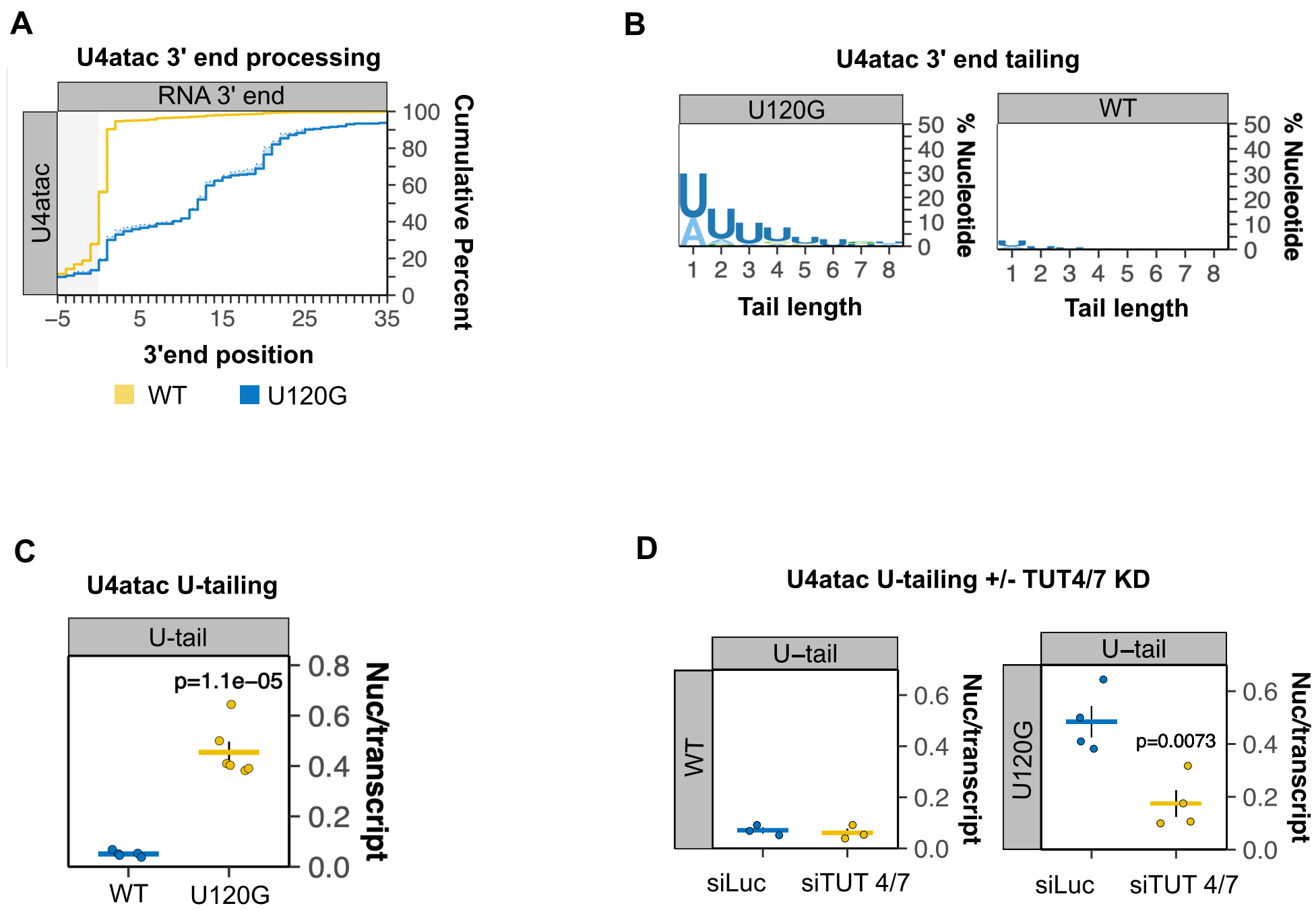

Supplementary Figure S8 related to Figure 7.
(A) Cumulative plot displaying cumulative fraction of 3’ end positions for U4atac WT (yellow) and U120G mutant (blue) snRNAs.
(B) Logo plots showing post-transcriptional tail compositions of U4atac WT and U120G mutant snRNAs.
(C) Average number of uridines observed in U4atac WT and U120G mutant snRNA post-transcriptional tails.
(D) Average number of uridines observed in U4atac WT (left) and U120G mutant (right) snRNA post-transcriptional tails in TUT4/7 knockdown (siTUT4/7) and control knockdown (siLuc) conditions.
